# Dopamine transporter blockade during adolescence increases adult dopamine function, impulsivity, and aggression

**DOI:** 10.1038/s41380-023-02194-w

**Published:** 2023-08-02

**Authors:** Deepika Suri, Giulia Zanni, Darshini Mahadevia, Nao Chuhma, Rinki Saha, Stephen Spivack, Nicolò Pini, Gregory S. Stevens, Annette Ziolkowski-Blake, Eleanor H. Simpson, Peter Balsam, Stephen Rayport, Mark S. Ansorge

**Affiliations:** 1 Department of Psychiatry, Columbia University, New York, NY 10032 USA; 2 Department of Developmental Neuroscience, New York State Psychiatric Institute, New York, NY 10032 USA; 3 Department of Molecular Therapeutics, New York State Psychiatric Institute, New York, NY 10032 USA; 4 Department of Neuroscience and Behavior, Barnard College, Columbia University, New York, NY 10032 USA

**Keywords:** Neuroscience, Molecular biology

## Abstract

Sensitive developmental periods shape neural circuits and enable adaptation. However, they also engender vulnerability to factors that can perturb developmental trajectories. An understanding of sensitive period phenomena and mechanisms separate from sensory system development is still lacking, yet critical to understanding disease etiology and risk. The dopamine system is pivotal in controlling and shaping adolescent behaviors, and it undergoes heightened plasticity during that time, such that interference with dopamine signaling can have long-lasting behavioral consequences. Here we sought to gain mechanistic insight into this dopamine-sensitive period and its impact on behavior. In mice, dopamine transporter (DAT) blockade from postnatal (P) day 22 to 41 increases aggression and sensitivity to amphetamine (AMPH) behavioral stimulation in adulthood. Here, we refined this sensitive window to P32-41 and identified increased firing of dopaminergic neurons in vitro and in vivo as a neural correlate to altered adult behavior. Aggression can result from enhanced impulsivity and cognitive dysfunction, and dopamine regulates working memory and motivated behavior. Hence, we assessed these behavioral domains and found that P32-41 DAT blockade increases impulsivity but has no effect on cognition, working memory, or motivation in adulthood. Lastly, using optogenetics to drive dopamine neurons, we find that increased VTA but not SNc dopaminergic activity mimics the increase in impulsive behavior in the Go/NoGo task observed after adolescent DAT blockade. Together our data provide insight into the developmental origins of aggression and impulsivity that may ultimately improve diagnosis, prevention, and treatment strategies for related neuropsychiatric disorders.

## Introduction

Neural plasticity during sensitive developmental periods endows organisms with the ability to adapt to their changing environment. As brain circuits form and mature, their use and activity provide instructive feedback to strengthen or weaken nascent neural connections [1]. These developmental windows of high plasticity are successful from an evolutionary perspective [1, 2]. However, sensitive period plasticity may also enable maladaptive shifts in ontogenetic pathways, resulting in heightened risk for pathological behaviors and neuropsychiatric disorders [3].

While monoamine systems are classically known for their modulatory roles in the mature brain, they also influence neurodevelopmental processes early in life [3], and can thereby alter ontogenetic pathways governed by sensitive period plasticity. A striking example is aggressive behavior resulting from lifelong hypofunction or ablation of monoamine oxidase A (MAOA) activity [4–7]. The behavioral phenotypes of genetic loss-of-maoa-function are conserved in mice and humans [5, 8]. However, chronic pharmacologic MAOA blockade during adulthood does not recapitulate these effects. The origins of heightened aggression in genetic *maoa* deficiency are in fact developmental and dopamine (DA)ergic in nature because the aggressive phenotype can be mimicked by transient developmental MAOA or DAT blockade from P22-P41 or by gestational intervention [4, 6]. Here, we refine the P22-41 period using narrower treatment windows.

Altered aggression in mice after P22-41 DAT blockade correlates positively with the locomotor response to amphetamine (AMPH) in adulthood [6]. In turn, increased behavioral response to AMPH is associated with a hyper-functioning DA system [9, 10]. Furthermore, striatal DA and DOPAC content is increased after peri-adolescent MAOA-blockade, and stimulation of VTA DAergic activity can trigger aggression [6]. Together these findings indicate that DAergic activity is altered as a consequence of peri-adolescent DAT blockade. Here, we test this hypothesis using slice and in vivo electrophysiology.

Aggression can result from enhanced impulsivity and cognitive dysfunction, and DA-signaling modulates both behavioral domains [11]. Moreover, adolescent amphetamine exposure can alter adult impulsive and aggressive behavior [12]. Our findings of increased behavioral response to amphetamine following peri-adolescent DAT blockade indicate that neural substrates regulating working memory and motivation might be affected. To investigate the behavioral consequences elicited by peri-adolescent DAT-blockade, we assessed impulsive behavior, cognition, motivation, and working memory. To investigate the link between altered DAergic activity in adulthood and impulsive behavior we studied the modulatory role of DA signaling using optogenetic stimulation in the Go/Nogo task.

Together, our findings reveal developmental mechanisms underlying increased aggression and impulsivity in mice. By extension, our data can inform how genetic and pharmacologic factors impacting DA signaling during peri-adolescence engender risk for aggressive and impulsive dysfunction in humans.

## Results

### DAT blockade between P32-41 increases aggression and AMPH responsivity in adulthood

**Fig. 1:**
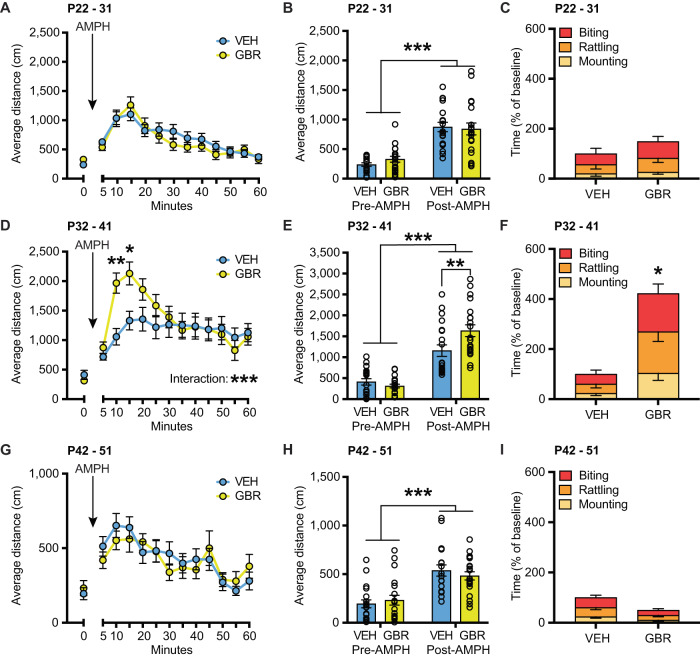
DAT blockade from P32-41, but not before or after, increases amphetamine-induced hyperlocomotion and baseline aggression in adulthood. Line graph showing the total distance traveled over time of mice administered GBR or VEH control from P22-P31 (*N* = 19 VEH, *N* = 20 GBR) (**A**), P32-41 (*N* = 20 VEH, *N* = 20 GBR) (**D**), and P42-51 (*N* = 19 VEH, *N* = 20 GBR) (**G**). The first bin (0) represents the average of behavior during the 5 min before amphetamine injection. The following bins (5–60) represent the average of behavior after amphetamine injection (0.5 mg/kg, i.p.). Upon amphetamine challenge, animals increased their total distance traveled. In the P32-41 treatment group, GBR animals had significantly higher locomotion after amphetamine challenge than VEH-treated animals (**D**). **B**, **E**, **H** Bar graph showing average distance traveled during 30 min pre-amphetamine (Pre-AMPH) and during 60 min post-amphetamine (Post-AMPH). **C**, **F**, **I** Bar graph showing the total time fighting as a percent of baseline. No effect of treatment was detected for P22-31 (*N* = 9 VEH, *N* = 9 GBR) (**C**) or P42-51 (*N* = 20 VEH, *N* = 19 GBR) (**I**). However, an effect of treatment was detected for P32-41 (*N* = 10 VEH, *N* = 10 GBR), with GBR treatment increasing aggressive behavior (**E**). *p < 0.05, **p < 0.01, ***p < 0.001.

To refine the DA-sensitive developmental window that affects adult aggression and AMPH responsivity we administered the DAT blocker GBR12909 (GBR) during three consecutive developmental periods: P22-P31 (pre-adolescence), P32-P41 (early adolescence), and P42-51 (late adolescence) [13–16]. We found no effect of GBR given during P22-P31 on adult amphetamine-induced hyperlocomotion or aggressive behavior (Fig. 1A–C). Analysis of ambulatory distance in the open field over time showed no significant interaction of treatment × time (*F*_(12, 444)_ = 1.365, *p* = 0.1794), a significant effect of time (*F*_(12, 444)_ = 28.51, *p* < 0.0001), and no significant effect of treatment (*F*_(1, 37)_ = 0.1730, *p* = 0.6798). Similarly, analysis of Pre-AMPH (30 min) and Post-AMPH (90 min) showed no significant interaction of treatment × time (*F*_(1, 37)_ = 0.7987, *p* = 0.3773), a significant effect of AMPH (*F*_(1, 37)_ = 70.02, *p* < 0.0001), and no significant effect of GBR (*F*_(1, 37)_ = 0.1504, *p* = 0.7004). GBR treatment from P22-31 did not alter aggression (*t*(16) = 0.7257, *p* = 0.4785). However, when GBR was administered during the peri-adolescent P32-P41 period, we found that adult animals displayed higher locomotion upon AMPH challenge as well as elevated aggression (Fig. 1D–F). Analysis of ambulatory distance traveled in the open field over time showed a significant interaction of treatment × time (*F*_(12, 456)_ = 5.459, *p* < 0.0001) and a significant effect of time (*F*_(12, 456)_ = 21.5, *p* < 0.0001). Post hoc analysis between treatments showed that GBR animals had significantly higher locomotion post-amphetamine at 5 min (*p* = 0.0019) and at 10 min (*p* = 0.0104). Likewise, analysis of Pre-AMPH and Post-AMPH showed a significant interaction of treatment × time (*F*_(1, 38)_ = 8.005, *p* = 0.0074) and a significant effect of time (*F*_(1, 38)_ = 103.4, *p* < 0.0001). Post hoc analysis revealed a significant effect of GBR compared to vehicle in the post-AMPH period (*p* = 0.0051). Moreover, GBR treatment from P32-41 increased aggressive behavior (*t*(18) = 2.856, *p* = 0.0105). GBR given during P42-P51 had no effect on adult amphetamine-induced hyperlocomotion or aggressive behavior (Fig. 1G–I). Analysis of ambulatory distance in the open field over time showed no significant interaction between treatment × time (*F*_(12, 444)_ = 0.8971, *p* = 0.5499), a significant effect of time (*F*_(12, 444)_ = 9.219, *p* < 0.0001), and no significant effect of treatment (*F*_(1, 37)_ = 0.03325, *p* = 0.8563). Analysis of Pre-AMPH and Post-AMPH showed no significant interaction of treatment × time (*F*_(1, 37)_ = 1.033, *p* = 0.3160, a significant effect of time (*F*_(1, 37)_ = 42.67, *p* < 0.0001), and no significant effect of treatment (*F*_(1, 37)_ = 0.03049, *p* = 0.8623). GBR treatment from P42-51 showed a trend for reduced aggressive behavior (*t* (37) = 1.763, *p* = 0.0909). Individual data for total time fighting as a percent of baseline and non-normalized data are plotted in Supplementary Fig. 1A–F. Analyzing the behavioral sub-components individually we find a significant increase in biting, mounting and tail rattling after GBR administration from P32-41 (Supplementary Table). We also find a trend decrease for mounting after GBR administration from P42-51 (Supplementary Table). For latencies, we detect a significant main effect of treatment only for the P32-41 period, where P32-41 GBR mice have shorter latencies to engage in fighting behavior when compared to VEH controls (Supplementary Fig. 1K). Again, we detect a trend for increased latencies for P42-51 GBR treated mice (Supplementary Fig. 1L). Analyzing bout frequencies for aggression behavior we find bi-directional consequences, where P32-41 GBR administration increased overall bout frequencies for biting, rattling, and mounting combined (main effect of treatment *F*_(1, 54)_ = 15.55, *p* = 0.0002) while P42-51 GBR administration reduced them (main effect of treatment *F*_(1, 111)_ = 5.188, *p* = 0.0247) (Supplementary Fig. 1N, O, respectively). Taken together, these data demonstrate the opening and closing of the DA-sensitive peri-adolescent P32-P41 window during which DAT blockade results in increased adult aggression and amphetamine-induced hyperlocomotion.

### P32-41 DAT blockade increases in vitro firing of DAergic neurons in adulthood

**Fig. 2:**
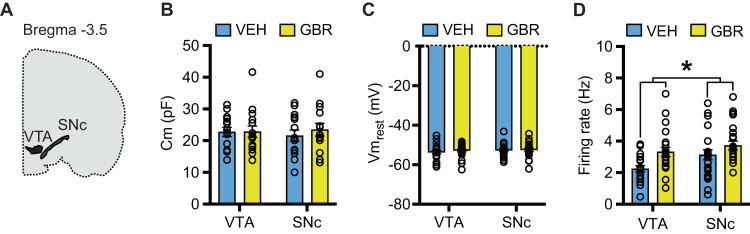
Peri-adolescent GBR administration increases in vitro DA neuron firing rates in adulthood. **A** Schematic of a coronal brain hemi-section containing the VTA and SNc. **B** Peri-adolescent GBR administration had no effect on membrane capacitance (*N* = 14 VEH, *N* = 15 GBR). **C** Increased in vitro firing rates of DA neurons in the VTA and SNc after P32-41 GBR administration (yellow), when compared to VEH (blue) (*N* = 19 VEH, *N* = 25 GBR). **D** Peri-adolescent GBR administration had no effect on resting membrane potential (*N* = 20 VEH, *N* = 25 GBR). *p < 0.05.

The enhanced effects of AMPH challenge on locomotion could be the consequence of potentiated pre-synaptic DA function. To investigate this hypothesis, we recorded from DAergic neurons in acute brain slice preparations. Specifically, mice were injected from P32-41 with GBR or VEH, brain slices were prepared after P60, and whole cell current clamp recording was performed in DAergic neurons in the ventral tegmental area (VTA) and the substantia nigra pars compacta (SNc) (Fig. 2A). Membrane capacitance (Cm) (Fig. 2B) and resting membrane voltage (Vm_rest_) (Fig. 2C) did not differ between GBR and VEH animals, neither in the VTA nor the SNc [membrane capacitance: no treatment × brain region interaction (*F*_(1, 53)_ = 0.2560, *p* = 0.6150) and no main effect of treatment (*F*_(1, 53)_ = 0.3193, *p* = 0.5744), resting membrane potential: no treatment × brain region interaction (*F*_(1, 85)_ = 0.1720, *p* = 0.6793), no main effect of treatment (*F*_(1, 85)_ = 0.4481, *p* = 0.5051)]. Even though these basic parameters were unaffected, we detected a main effect of treatment on firing rates (effect of treatment: *F*_(1, 84)_ = 8.686, *p* = 0.0042) with GBR treatment increasing overall DAergic firing rates. While we did not detect an interaction between treatment and brain region (treatment × brain region: *F*_(1, 84)_ = 0.7038, *p* = 0.4039), the mean effect size was higher in the VTA than in the SNc (VTA_GBR/VEH_ = 1.47 Hz, SNc_GBR/VEH_ = 1.19) (Fig. 2D).

### P32-41 DAT blockade increases in vivo firing of putative DAergic neurons in the adult

**Fig. 3:**
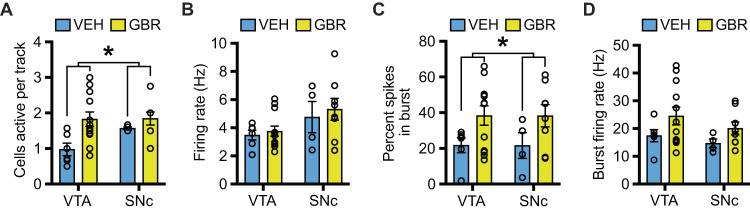
Peri-adolescent GBR administration increases in vivo DA neuron cell activities. P32-41 GBR administration increases the number of active cells per track. **A** as well as the percent of spikes occurring in bursts (**C**). P32-41 GBR administration does not alter overall firing rates (**B**) or burst firing rates (**D**). (*N* = 6 VEH_VTA_, *N* = 4 VEH_SNc_, *N* = 12 GBR_VTA_, *N* = 8 GBR_SNc_). *p < 0.05.

Next, we assessed DAergic neuron firing in vivo by recording from putative DAergic neurons in the VTA and SNc of anesthetized mice. Analysis of the cells active per track showed a significant main effect of treatment (*F*_(1, 26)_ = 6.387, *p* = 0.00179) and no significant effect of brain region (*F*_(1, 26)_ = 1.872, *p* = 0.1829) nor treatment × brain region interaction (*F*_(1, 26)_ = 1.649, *p* = 0.2104), with GBR treatment increasing the number of active cells (Fig. 3A). Analysis of DAergic neuron firing rates showed no significant main effect of treatment nor treatment × brain region interaction (*F*_(1, 26)_ = 0.4225, *p* = 0.5214 and *F*_(1, 26)_ = 0.04641, *p* = 0.8311, respectively, Fig. 3B). Analysis of DAergic neuron firing patterns revealed a main effect of treatment for percent of spikes in bursts (*F*_(1, 26)_ = 6.398, *p* = 0.0178, Fig. 3C) and a trend for a main effect of treatment on burst firing rate treatment (*F*_(1, 26)_ = 3.542, *p* = 0.0711, Fig. 3D). Using cells as N we also found a main effect of treatment for percent of spikes in bursts: (*F*_(1, 171)_ = 6.634, *p* = 0.0109, Supplementary Fig. 2C). No significant treatment × brain region interaction was detected for percent of spikes in bursts (*F*_(1, 26)_ < 0.0001, *p* = 0.9969) nor burst firing rate (*F*_(1, 26_ = 0.06487, *p* = 0.8010). Even though we did not detect significances for treatment × brain region interaction for any of the parameters, the treatment effect sizes are consistently larger in the VTA than SNc.

### P32-41 DAT blockade does not affect working memory and reversal learning in adults

**Fig. 4:**
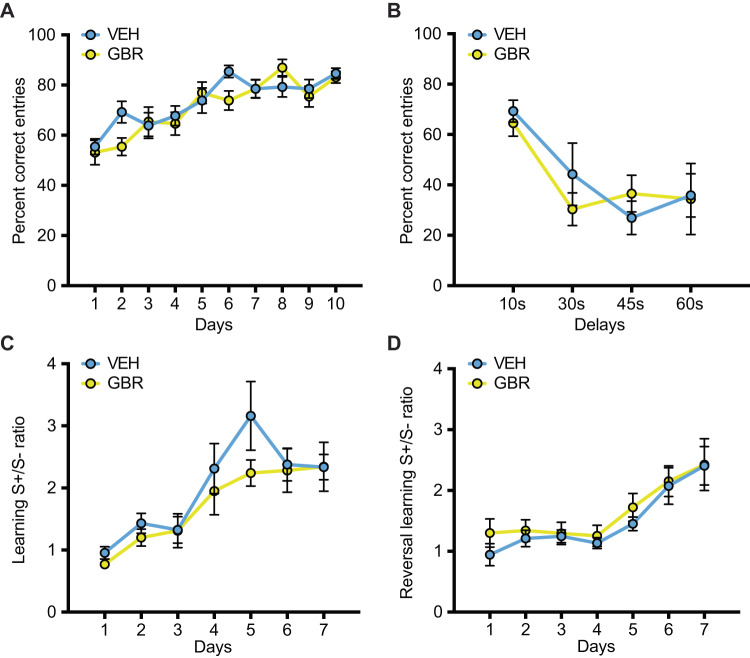
Peri-adolescent GBR treatment does not affect cognition in the T-maze and a reversal learning operant task. **A** In the T-maze, P32-41 GBR administration does not alter the percent of correct arm entries over 10 days of training. **B** Increasing delays reduced the percent of correct arm entries with no effect of P32-41 GBR administration (*N* = 15 VEH, *N* = 14 GBR). In an operand reversal learning task, P32-41 GBR administration does not alter the discrimination ratio between rewarded stimuli (S^+^) and non-rewarded stimuli (S^−^) over 8 days of initial training. **C** or during 8 days following rule reversal (**D**) (*N* = 13 VEH, *N* = 14 GBR). *p < 0.05.

Aggression can result from a lack of top-down cortico-limbic control in brain structures that are relevant to working memory and cognitive flexibility [17]. Owing also to the role of DA signaling in these behavioral domains [18, 19], we examined if the origin of aggression after DAT blockade during peri-adolescence could be related to working memory and cognitive inflexibility. We found that in the non-match to sample T-maze task learning abilities were unchanged. No effect of treatment nor treatment × days interaction was detected for percent correct arm entries (treatment: *F*_(1, 24)_ = 0.8821, *p* = 0.3570; treatment × days interaction: *F*_(9, 216)_ = 1.419, *p* = 0.1813). A significant effect of days demonstrated learning (*F*_(9, 216)_ = 13.75, *p* < 0.0001) (Fig. 4A). Likewise, there was no effect of treatment in percent correct arm entries at different delays (*F*_(1, 25)_ = 0.2066, *p* = 0.6533) nor treatment × delay interaction (*F*_(3, 65)_ = 0.7816, *p* = 0.5085). A significant effect of delay demonstrated an equal decrease in performance with an increase in task difficulty in both VEH and GBR animals (*F*_(3, 65)_ = 8.705, *p* < 0.0001) (Fig. 4B). Next, we tested animals in an operant reversal learning task, which assesses the ability to discriminate between two stimuli, one rewarded and one not rewarded. Treatment did not affect the acquisition phase of the discrimination task as there was no significant treatment × days interaction (*F*_(6, 153)_ = 0.6957, *p* = 0.6535) and no significant effect of treatment (*F*_(1, 26)_ = 1.201, *p* = 0.2832). A main effect of days demonstrated discrimination learning over time (*F*_(6, 153)_ = 12.37, *p* < 0.0001) (Fig. 4C). After successful discrimination learning we tested mice over 8 days with rule reversal. Treatment did not affect reversal learning given that there was no treatment × days interaction (*F*_(6, 155)_ = 0.1631, *p* = 0.9861) and no effect of treatment (*F*_(1, 26)_ = 1.074, *p* = 0.3097). Again, a main effect of days demonstrated reversal learning over time (*F*_(6, 155)_ = 10.89, *p* < 0.0001) (Fig. 4D). These results suggest that P32-41 DAT blockade did not affect learning, working memory and cognitive flexibility.

### P32-41 DAT blockade increases adult impulsivity

**Fig. 5:**
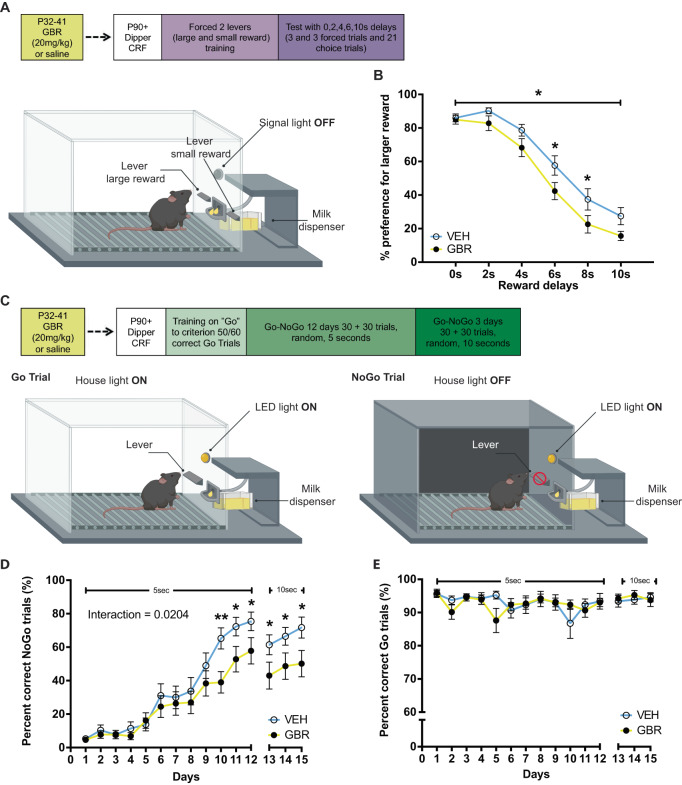
Peri-adolescent GBR treatment increases impulsivity in the delayed discounting and Go/NoGo tasks. **A **Experimental design and schematic for delayed discounting task testing. Animals were trained to associate one lever with a large reward and a second lever with a smaller reward. Animals were then presented with both levers to freely choose between levers with increasing delays associated with the larger reward. **B** Percent preference for the larger reward decreased with increasing delays. P32-41 GBR administration resulted in steeper discounting of the larger reward with an increase in delay when compared to P32-41 VEH administration (*N* = 13 VEH, *N* = 14 GBR). **C** Experimental design and schematic for Go/NoGo task testing. Animals first underwent dipper training, followed by continuous reinforcement (CRF) training until criterion (50/60 correct Go trials). NoGo trials were introduced after criterion was met for 3 consecutive days. 30 Go and 30 NoGo trials were randomly presented for 5 s for 12 days. Go trials used house light on and lever LED light off as cues (cartoon lower left), vice versa NoGo trials used light house off and lever LED light on (cartoon lower right). From day 13 onwards the duration of Go and NoGo trials was extended to 10 s. **D** All animals learned to inhibit behavioral responses as revealed by increasing percent correct NoGo responses over time. However, P32-41 GBR administration impaired performance as the increase in percent correct NoGo responses over time was less steep. **E** Go trial performance was not affected by either time or drug treatment. (*N* = 19 VEH and *N* = 21 GBR). *p < 0.05.

Aggression and impulsivity behaviors are highly related [20, 21]. Impulsivity can manifest as a lack of behavioral inhibition (impulsive actions) or as a decrease in tolerance for delayed rewards (impulsive choices) [22–24]. Both behaviors are regulated by the DAergic system [25]. Thus, we sought to examine if, in parallel with adult aggressive behavior, adult impulsivity was also sensitive to P32-41 DAT blockade. Impulsive choice was examined using the Delayed Discounting task, which assesses the ability to tolerate reward-associated delays (Fig. 5A, B). Indeed, we found a significant effect of treatment (*F*_(1, 40)_ = 4.516, *p* = 0.0398), with GBR mice showing increased preference for small but more immediate rewards. A main effect of delay duration (*F*_(5, 200)_ = 158.6, *p* < 0.0001) demonstrates that both treatment groups are sensitive to increasing delays. While we did not detect a significant treatment × delay duration interaction (*F*_(5, 200)_ = 1.404, *p* = 0.2242), post hoc comparisons were significant only for 6 s and 8 s delays (Fig. 5B). Next, we examined impulsive actions using the Go/No-Go task, in which the ability to refrain from responding during specific trials is rewarded [24, 25] (Fig. 5D, E). We find that animals learned to inhibit behavioral responses over time by increasing the percent correct NoGo responses, but GBR treated animals performed worse than VEH controls with task progression (Fig. 5F). In the 5 s NoGo trials (days 1–12), we detected a treatment × days interaction (*F*_(11, 406)_ = 2.085, *p* = 0.0204) and a significant effect of days (*F*_(11, 406)_ = 50.66, *p* < 0.0001). Post hoc analysis revealed that GBR treated mice have a slower learning curve for percent correct NoGo trials when compared to VEH-treated controls with significant differences at days 10 to 12. All animals decreased their percent correct NoGo trials when NoGo trial length was increased to 10 s (days 13–15). Within days 13–15 all mice increased their performance (significant effect of days: *F*_(2, 74)_ = 3.801, *p* = 0.0268), but GBR treated mice had lower correct percent NoGo trials when compared to VEH controls (significant effect of treatment: *F*_(1, 37)_ = 4.356, *p* = 0.0438). Go trials were not affected by treatment throughout the experiment (no treatment × days interaction for days 1–12: *F*_(11,418)_ = 1.548, *p* = 0.1119, no treatment effect for days 1–12: *F* = _(1, 38)_ = 1.994, *p* = 0.1660, no treatment × days interaction for days 13–15: *F*_(2.76)_ = 0.5511, *p* = 0.5786, and no treatment effect for days 1–12: *F*_(1, 38)_ = 1.605, *p* = 0.2129; Fig. 5G). These data suggest that P32-41 DAT blockade increased both types of impulsivities in adults.

### P32-41 DAT blockade does not affect motivation

**Fig. 6:**
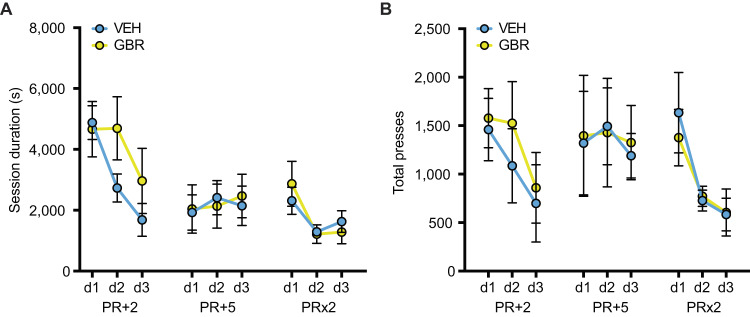
Peri-adolescent GBR treatment does not change motivation in a progressive ratio task. P32-41 GBR treatment does not alter performance in progressive ratio tasks as assessed by session duration (**A**) and total presses during a session (**B**) for ratio schedules of plus 2 (PR + 2), plus 5 (PR + 5), and times 2 (PR × 2) (*N* = 9 VEH and *N* = 8 GBR). *p < 0.05.

To test for a potential contribution of motivation in performing the impulsivity tasks, we assessed behaviors of the two treatment groups on the progressive ratio task. This control experiment is particularly important considering the role of dopamine in mediating behavioral motivation [26]. Importantly, we found no significant effect of treatment on performance in the progressive ratio task. In session duration (Fig. 6A) over different progressive ratios (PR + 2, PR + 5, and PR × 2) there was no significant treatment × days interaction (*F*_(8, 107)_ = 0.8756, *p* = 0.8356), nor a significant effect of treatment (*F*_(1, 15)_ = 0.3543, *p* = 0.5606). A significant effect of days (*F*_(8, 107)_ = 7.754, *p* < 0.0001) demonstrated learning and sensitivity to workload. In total presses (Fig. 6B) over different progressive ratios (PR + 2, PR + 5, and PR × 2), again there was no significant treatment × days interaction (*F*_(8, 103)_ = 0.1102, *p* = 0.9988) and no effect of treatment (*F*_(1, 15)_ = 0.01035, *p* = 0.9203). A main effect of days (*F*_(8, 103)_ = 2.449, *p* = 0.0181) again demonstrated learning and sensitivity to workload. These data suggest that P32-41 DAT blockade does not affect motivation to perform a task.

### Optogenetic stimulation of VTA DAergic neurons increases impulsivity

**Fig. 7:**
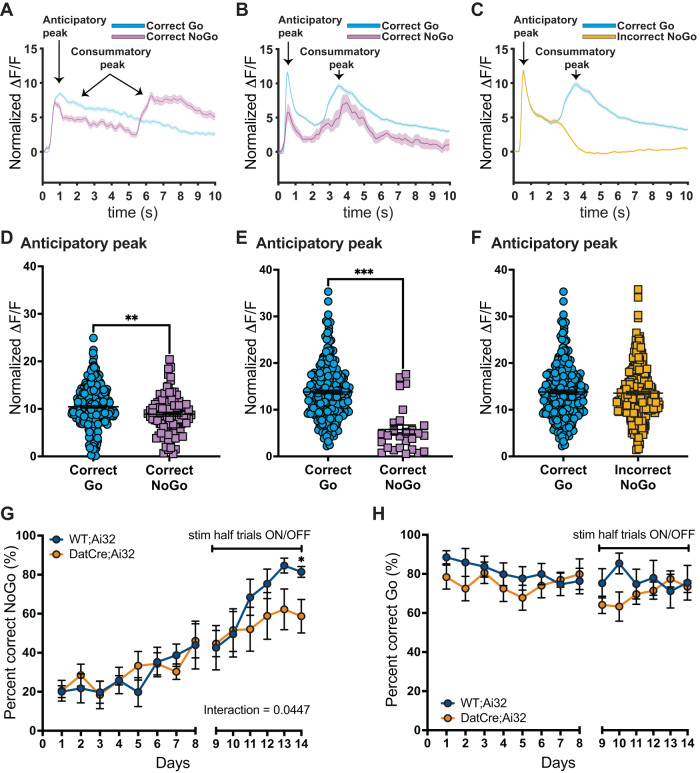
Optogenetic stimulation of VTA dopamine neuronal activity increases action impulsivity. **A** Normalized dopamine activity (dF/F) aligned to trial start (lever presentation) during the Go-NoGo task, separated for correct Go (cyan) and correct NoGo (magenta) trials. The first dopamine peak corresponds to the anticipatory peak followed by the consummatory peak, as indicated by arrows. Go trials had no delay between lever press and dipper out, while NoGo trials had 5 s delay. The anticipatory DA peak averages of Correct Go trials is larger than the average anticipatory DA peak of the Correct NoGo trials (**D**). **B**, **C** Normalized dopamine activity (dF/F) aligned to trial start (lever presentation) during the Go-NoGo task, with 4 s delays between lever press and dipper out for both Go and NoGo trials. The anticipatory DA peak average of Correct Go trials is larger than the average anticipatory DA peak of Correct NoGo trials (**E**). The anticipatory DA peak average of Correct Go trials is not different from the average anticipatory DA peak of Incorrect NoGo trials (**F**). **G** Prior to optogenetic stimulation of VTA DAergic neuronal activity (days 1–8), mice learn to inhibit behavioral responses as revealed by increasing percent correct NoGo responses over time with no effect of genotype. However, with optogenetic stimulation of VTA DAergic neuronal activity (days 9–14), performance on NoGo trials is impaired. **H** Go trial performance was not affected by either time or optogenetic stimulation of VTA DAergic neuronal activity, *N* = 6 WT;Ai32, *N* = 7 DatCre;Ai32. *p < 0.05, **p < 0.01, ***p < 0.001.

DA neuron signaling aligns with the value of a reward, either expected or delivered [27, 28], and it is necessary for learning the association of a stimulus paired to a reward [29]. During the learning phases of a reward-based Go/NoGo task DAergic activity scales with the encoding of the reward predictive stimulus [30]. Here we measured VTA DAergic neuron activity by measuring Ca^2+^ transients in vivo during behavior. We injected an AAV (AAV1.Syn.Flex.GCaMP6s.WPRE.SV40) virus expressing the calcium (Ca^2+^) sensor GCaMP6s into the VTA of heterozygous Dat^IRESCre^ mice. Using fiber photometry, we recorded fluorescence signals from 2 mice during the Go/NoGo task. For Go trials, VEH and GBR animals took on average 1.2 s to press the lever (no treatment effect, *t* = 6.558, *p* = 0.5219) (Supplementary Fig. 3A), and 0.4 s to consume the reward (no treatment effect, *t* = 1.939, *p* = 0.0683) (Supplementary Fig. 3B). We therefore aligned the analysis of the fluorescent signals to the lever extension and confirmed DAergic neuronal activity during the anticipatory phase (1 s after lever extension) as well as during reward consumption (1.5 s after lever extension in correct Go trials and 5 s after lever extension in correct NoGo trials) (Fig. 7A and Supplementary Fig. 4A–C). Of note, the peak during the first 3 s of lever extension was higher for correct Go trials than for correct NoGo trials (*t* = 3.241, *p* = 0.0013, Fig. 7D). To better separate the anticipatory peak from the consummatory peak for both trial types, we also ran a second version of the task where both Go and NoGo trials have the same delay (4 s) between lever extension and reward (Fig. 7B and Supplementary Fig. 4G–I). Again, we find that the peak at lever extension was higher for correct Go trials than for correct NoGo trials (*t* = 7.024, *p* < 0.0001, Fig. 7E). Furthermore, incorrect NoGo trials show the same high amplitude of the anticipatory peak as the Correct Go trials (Fig. 7C, F). Lastly, incorrect NoGo trials furthermore lack the consummatory peak due to the lack of positive reinforcement (no milk), and even display a steeper decline (inverted S-shape), akin to a negative prediction error response, typical for VTA DAergic neurons (Fig. 7C). Based on the DAergic activity signatures during the anticipatory phase, we predicted that elevated DAergic neuron activity in GBR treated mice drives impulsive behavior causing mice to respond with Go-responses in NoGo-trials. To test this hypothesis, we performed a DAergic gain-of-function experiment to mimic the GBR phenotype using in vivo optogenetics [6, 31]. Specifically, we implanted optic fiber in the VTA of DatCre;Ai32 mice that express channelrhodopsin (ChR2) in DAergic neurons and Wt;Ai32 littermate control mice that do not express ChR2. To test for placement, we evaluated behavior in the open field, where stimulation over multiple minutes increases locomotion [32, 33]. Indeed, we found that in vivo, optogenetic stimulation with 3 min of laser pulses (473 nm, 10 ms, 20 Hz) increased locomotor activity of DatCre;Ai32 but not WT;Ai32 mice (genotype × stimulation interaction: *F*_(3, 144)_ = 16.27, *p* < 0.0001, Supplementary Fig. 5A). Post hoc analysis showed that during light ON periods DatCre;Ai32 mice were more active than Wt;Ai32 animals (*p* < 0.0001), supporting correct targeting.

For operant behavior, all animals were left unstimulated until and including day 8. DatCre;Ai32 and Wt;Ai32 acquired the NoGo task equally well. Starting on day 9 we delivered 3 s of light pulses (20 Hz, 10 ms, 8–10 mW) aligned with lever presentation (1-s prior lever out and 2 s into lever out). Optogenetic stimulation resulted in a lower percent correct NoGo trials in DatCre;Ai32 mice when compared to Wt;Ai32 mice, supporting our hypothesis that elevated VTA DAergic activity at trial signaling drives impulsive choices (Fig. 7G). Repeated measure two-way ANOVA of day 1 to 8 (no stimulation) revealed no interaction of genotype × days (*F*_(7, 77)_ = 0.5611, *p* = 0.7852), a significant effect of days (*F*_(7,77)_ = 4.084, *p* = 0.0007), and no significant effect of genotype (*F*_(1, 11)_ = 0.09306, *p* = 0.7660). Two-way ANOVA of the stimulation days 9–14 revealed a significant interaction of genotype × stimulation (*F*_(5, 55)_ = 2.452, *p* = 0.0447), a significant effect of stimulation (*F*_(3.118, 34.30)_ = 10.21, *p* < 0.0001), and no significant effect of genotype (*F*_(1, 11)_ = 1.100, *p* = 0.3168). Post hoc multiple comparison Fisher’s LSD test revealed that on day 14 DatCre;Ai32 animals had significantly fewer percent correct NoGo trials compared to Wt;Ai32 animals (*p* = 0.0392). For Go-trials, we found no interaction of genotype × days (*F*_(13, 137)_ = 1.101, *p* = 0.3634), no effect of days (*F*_(13,137)_ = 1.191, *p* = 0.2919), and no effect of genotype (*F*_(1, 11)_ = 1.151, *p* = 0.3062) (Fig. 7H). Also for Go trials, we found no effect of optogenetic stimulation on the latency to lever press (Supplementary Fig. 6A), or the latency to consume the reward (Supplementary Fig. 6B). Taken together, our data demonstrate that increased activity of VTA DAergic neurons during reward predictive stimulus presentation promotes impulsive choice without altering performance on Go-trials, mimicking the behavior of GBR mice.

### Optogenetic stimulation of SNc DAergic neurons impairs response on Go-trials but does not alter action impulsivity

**Fig. 8:**
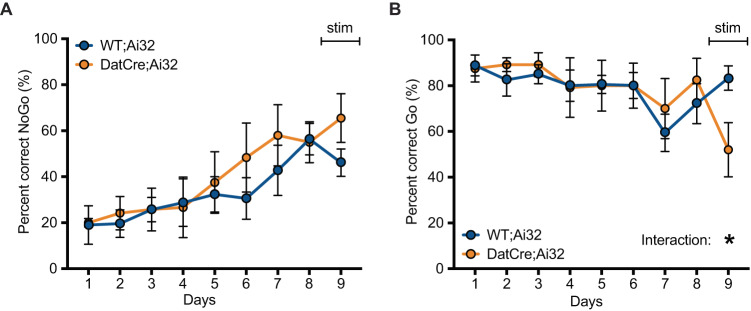
Optogenetic stimulation of SNc dopamine neuronal activity impairs performance on Go trials but does not alter action impulsivity. **A** Prior to optogenetic stimulation of SNc DAergic neuronal activity (days 1–8), mice learn to inhibit behavioral responses as revealed by increasing percent correct NoGo responses over time with no effect of genotype. Performance on NoGo trials was not affected by optogenetic stimulation of SNc DAergic neuronal activity (day 9). **B** Go trial performance was not affected by either time, however optogenetic stimulation of SNc DAergic neuronal activity decreased performance as measured by percent correct Go trials, *N* = 9 WT;Ai32, *N* = 6 DatCre;Ai32. *p < 0.05.

SNc DAergic neuronal activity is pivotal in movement coordination and reward [34, 35] and more importantly regulates the suppression of movement initiation during learning [36]. Therefore, we investigated the effect that optogenetic DA cell stimulation targeted to the substantia nigra compacta (SNc) has on the Go/NoGo task. In vivo optogenetic stimulation of DatCre;Ai32 mice through an optical fiber implanted in the SNc showed that brief 3 s of 473 nm laser pulses 10 ms long did not alter the percent correct NoGo trials and all animals increased the percent correct of NoGo trials over time irrespective of the genotype (Fig. 8A). Two-way ANOVA revealed no interaction of genotype × days (*F*_(8, 91)_ = 0.5855, *p* = 0.7875), a significant effect of days (*F*_(4.303, 4895)_ = 6.732, *p* = 0.0002), and no significant effect of genotype (*F*_(1, 13)_ = 0.6361, *p* = 0.4395). Analysis of the percent correct Go trials revealed that SNc dopamine cell stimulation significantly impaired task performance with a decrease of the percent correct Go trials (Fig. 8B). Two-way ANOVA revealed a significant interaction of genotype × stimulation (*F*_(8, 89)_ = 2.373, *p* = 0.0230), a significant effect of stimulation (*F*_(3.704, 41.21)_ = 3.896, *p* = 0.0104), and no significant effect of genotype (*F*_(1, 13)_ = 0.01933, *p* = 0.8916). Post hoc analysis revealed that at day 9 of stimulation DatCre;Ai32 animals had a significantly decreased percent correct Go compared to Wt;Ai32 (*p* = 0.0462). These data suggest that the impulsivity observed after P32-41 DAT blockade results from increased activity of VTA rather than SNc DA neurons.

## Discussion

Sensitive periods are essential for experience-dependent refinement of neural circuits [1]. These developmental windows are characterized by heightened brain plasticity. We identified P32-41 as a DA-sensitive developmental period during which DAT blockade increases aggression, impulsivity, and amphetamine responsivity in adulthood. Altered behavior is associated with increased DAergic neuronal activity, and elevated VTA DAergic activity drives impulsive choices.

Because we did not observe robust changes in adult behavior after P22-31 or P42-51 DAT blockade, the systems that are underlying aggression and behavioral amphetamine response are likely maturing with peaking plasticity during P32-41. Indeed, DA system maturation is ongoing during this time window [13, 37, 38], when the cortex undergoes progressive DAergic innervation [39], DAT increases in density [40], DA receptors are pruned [41, 42] in particular in NAc [43], and DAergic activity peaks [44, 45]. Therefore, we hypothesize that auto-regulatory processes during P32-41 lead to permanently changed set points in DA function relevant for aggression and amphetamine response. Given that P32-41 DAT blockade also increases impulsivity, we furthermore hypothesize that the DA function underlying this behavioral domain is also permanently altered. In contrast, we find no effects of P32-41 DAT blockade on baseline locomotor activity, motivation, working memory, and reversal-learning—behaviors known to be modulated and controlled by DAergic activity. Hence, we conclude that DAergic subsystems related to these behavioral domains are either not affected (they don’t transition through a P32-41 sensitive period), or changes in DAergic function are too subtle to affect these behaviors. One interesting observation is that aggression might be slightly reduced after P42-51 GBR exposure, which indicates that sensitive periods might be concatenated and bi-directional. Another interesting comparison is that amphetamine administration from P22-31 but not P35-44 also impairs behavioral inhibition [46]. This finding demonstrates the robust nature of this sensitive period but also highlights that genetic mouse background and/or specific pharmacology may influence sensitive period boundaries. Further mapping of behavioral consequences and refining of (the) developmental window(s) will allow us to formulate more specific predictions as to which DAergic subsystems are affected.

Starting to test our hypothesis of altered adult DAergic activity resulting from P32-41 DAT blockade, we performed electrophysiology experiments. In vitro, we found that DAergic neurons indeed exhibit increased spontaneous firing rates after peri-adolescent DAT blockade. Firing in slices is largely determined by intrinsic ion channel properties, which determine pacemaker firing as well as baseline excitability [47, 48]. In vivo, we do not find an effect of peri-adolescent DAT blockade on tonic firing. Tonic DAergic neuron activity is largely controlled by tonic GABAergic input [49]. Thus, our findings indicate that this GABAergic control is not altered. However, we found an increased number of VTA DAergic neurons active as well as increased bursting activity after peri-adolescent DAT blockade. Both parameters are strongly dependent on glutamate and nicotine input [50–52]. Thus our in vitro and in vivo data together indicate that peri-adolescent DAT blockade renders DAergic neurons more sensitive to phasic excitatory input. This interpretation can explain the lack of behavioral effects in baseline locomotor activity and motivation. Yet, if more cells are readily activated by specific triggers and their firing patterns favor bursting, this can lead to significantly increased DAergic release in response to phasic excitatory input [53]. Behavioral phenotypes indicate that this increased sensitivity is DAergic pathways specific, preferentially affecting mesolimbic and mesocortical pathways over the nigrostriatal pathway. Although we did not detect statistically significant interactions when stratifying our electrophysiological data by brain region (VTA vs. SNc), effect sizes in the VTA were consistently larger when compared to the SNc. This conclusion is also in line with AMPH sensitivity being a behavioral indicator of an over-responsive VTA DA system [54–56]. Future experiments using in vivo cyclic voltammetry or genetically encoded DA sensors will directly assess pathway activity and heightened responsiveness (via increased sensitivity to excitatory input) in vivo. It is possible that multiple pre-synaptic aspects combined will produce prominent DA release net effects selectively in specific DAergic pathways. If for example more DAergic neurons are active and their burst-firing properties are increased (as we have found), and in addition, their DA-release capacity is increased, these parameters might synergize to significantly increase DA release during specific behaviors.

It is a telling coincidence that behaviors sensitive to P32-41 DAT blockade are also the ones that are still maturing during this same period. Play behavior during adolescence forms adult agonistic behavior and social peer interactions [1, 57–59]. Exploratory risk-taking and motivation during adolescence form adult experience-based risk/reward preferences and impulsive behaviors [60, 61]. Together, these processes play a pivotal role in the establishment of adaptive behaviors [57–64], and they are necessary for a normal transition into adulthood with behavior that is adapted to the social and environmental context [65, 66]. This reflects the sensitive period principals highlighting ethological footings. Although the role of DA in adolescent behaviors has been investigated [9, 67], the impact of behavior and experience during adolescence and associated long-term effects on DA-modulated behaviors in the adult are not well understood. In other words, how does DAergic brain circuitry adapt to behavioral and experience-based feedback? In an elegant study that combined ethological and molecular approaches in the study of DAergic pathway maturation it has for example been shown that microglia-mediated D1-receptor pruning in the NAc is required for natural changes in male social play behavior during adolescence [68]. Further understanding the dynamic feedback of behavior on such processes will be critical to improve our understanding of sensitive periods.

Aggression is correlated with and can result from cognitive dysfunction and enhanced impulsivity [69–71]. DA-signaling modulates impulsivity and cognition [24, 72, 73]. Furthermore, altered behavioral response to AMPH after P32-41 DAT blockade indicates that neural substrates regulating working memory and reversal learning might be affected [74–76]. Yet, we report no effect of GBR on working memory nor reversal learning, dissociating these behavioral domains by developmental period sensitivity. However, we find increased impulsivity after GBR, supporting a strong mechanistic relationship with aggression, and even pointing at shared ontogeny and etiology. Aggression can be triggered by stimulation of VTA DAergic neurons [6]. To investigate the mechanistic relationship between aggression and impulsivity, we tested if impulsivity is likewise sensitive to DAergic activation. Indeed, we find that optogenetic stimulation of VTA but not SNc DAergic neurons increases impulsivity in the Go-NoGo task. This finding is in line with a role for the nucleus accumbens in impulsive action [77–79], and specifically with increased impulsivity in the 5-choice serial reaction time task after optogenetic stimulation of the VTA to nucleus accumbens shell pathway [80]. This finding is also in line with DA projections to the PFC, which continue to grow and mature across adolescence, playing a role in impulsive action [81–83]. We had based our optogenetic stimulation protocol on our fiber photometry data that had revealed a reduced anticipatory peak in VTA DAergic activity for correct NoGo trials. We thus had speculated that we can drive impulsive choices by stimulating VTA DAergic activity during lever presentation. While our hypothesis was confirmed, we want to highlight that our fiber photometry data were collected from only two mice. A more detailed analysis of endogenous activities and coding properties of DAergic neurons can only be done with a larger dataset. Further analyses of endogenous DAergic pathway activities during behavior and their necessary and sufficient roles for aspects of aggression and impulsive control will allow us to more fully understand how closely related the underlying mechanisms of these behaviors are, and how selectively targetable treatment approaches for specific dysfunctions might be.

Aggression can be behaviorally classified into reactive aggression which occurs impulsively in response to perceived external threat and proactive aggression that is premeditated, planned, and directly motivated by a drive for appetitive reward [84–86]. While we found strong effects of P32-41 DAT blockade on aggression and impulsivity, we did not find effects on motivation as assessed by the progressive ratio task. This finding indicates that the P32-41 sensitive period may be specific for reactive aggression without affecting appetitive aspects of aggression, potentially isolating stress-response-related behavior. Alternatively effects on the DAergic reward pathways might be too subtle to impact progressive ratio performance but may be unmasked in the context of aggression. This hypothesis can be directly tested by assessing the impact of P32-41 DAT blockade on the appetitive drive for aggressive behavior.

Our findings also agree with human vulnerabilities to impulsivity and aggression conferred by functional genetic polymorphisms that act during development [3]. Our data also indicate that the specific environmental factor “drug exposure” could alter risk for pathological aggression, impulsivity, and potentially substance use disorder. Drugs that target the DA systems are commonly prescribed during adolescence for attention deficit disorders [87–89]. Use of stimulant medications without prescription is also prevalent—largely for recreational use or to improve performance in high school/college [90–92]. While these drugs are generally considered safe without severe side effects, their long-lasting consequences are not fully understood. Our experiments using mice potentially provide insight into the consequences of transient stimulant exposure during early adolescence in humans. However, it is important to note that our experiments were performed in wild-type animals and not in animals that display phenotypes resembling ADHD symptomatology. Hence, we cannot directly translate our findings to the clinically appropriate use of psychostimulants, but maybe more so to chronic recreational use or improper prescription (over-prescription). In a diseased state that results from dopamine system hypofunction, transient exposure to psychostimulants during adolescence might potentially be corrective, but this hypothesis needs to be experimentally tested. Critically, we argue that an understanding of the underlying biology is necessary for a clear risk/benefit evaluation of recreational or therapeutic drug exposure prior to adulthood is impeded.

In summary, deficits in impulse control and increased aggression are prominent symptoms of mental disorders such as attention deficit disorders, schizophrenia, bipolar disorder, and substance use disorders. Our data expand the knowledge of sensitive periods that determine the developmental trajectory of neuronal pathways underlying impulsive and aggressive behavior. This insight is a necessary step toward improving diagnosis, prevention, and treatment approaches for pathological and maladaptive behavior.

## Methods

### Subjects behavior, and electrophysiology

Mice (129SvEv/Tac) were bred at Columbia Psychiatry, New York State Psychiatric Institute. Only male mice were used to examine aggression behavior and amphetamine response. Male and female mice were used to assess operant behavior, working memory, and electrophysiology. No interactions between sex other independent variables were detected, and data were collapsed for sex as a consequence. Behavior, slice electrophysiology, and in vivo electrophysiology were performed as previously described [6, 32, 93, 94] and as detailed in the Supplementary Material. Animal testing was conducted in accordance with the Principles of Laboratory Animal Care National Institute of Health (NIH) guidelines and the institutional animal committee guidelines.

### Optogenetics and fiber photometry

For optogenetics, fiber optic implants were prepared using 1.25 mm zirconia ferrules (Precision Fiber Products) with a 200 µm optical fiber (ThorLabs). At 3 months of age fiber-optic implants were placed targeting the VTA (anterior/posterior (AP) −3.5 mm, medial/lateral (ML) −0.5 mm, and dorsal/ventral (DV) −3.5 mm), and SNc (AP −3.5, ML −1.5, DV −5.5). All coordinates are in reference to Bregma (AP and ML) and brain surface (DV). Mice were allowed to recover for at least 2 weeks after surgery.

For fiber photometry, Dat^IRESCre^ mice were stereotaxically injected in the VTA ((AP) −3.5 mm, (ML) −0.5 mm, and (DV) −3.5 m) with a GCaMP6 virus (AAV1.Syn.Flex.GCaMP6s.WPRE.SV40) and implanted with a 2.5 mm flat tip metal 400 µm optic fiber patch cord (MFC_400/430-0.48_5mm_ MF2.5_FLT, Doric Lenses Inc.). We waited at least 6 weeks prior to recording to allow for viral expression. For recording, we used dual excitation wave-length fiber photometry and corrected the channel (470 nm) with a reference point of tissue-auto-fluorescence near its isosbestic point (405 nm) (Thorlabs Inc.). We used Synapse software (TDT) to acquire the data. DAergic activity was recorded during the Go/No-Go task and a TTL trigger (Med Associates Inc., St Albans, VT) enabled the alignment of the lever contingencies of the operant task to the DAergic brain activity. Data were analyzed with custom-written MATLAB scripts (see Supplementary Material for details).

After completion of experiments, animals with intact implants were perfused transcardially with NaCl 0.9% and PFA 4%, and brains were sectioned and checked for anatomical fiber placement (Supplementary Fig. 7). Missing placement validation was due to attrition.

### Statistical analysis

Statistical analyses were performed using Prism GraphPad version 9.3.1 software, LLC. Statistical analyses of main effects and interactions were performed using *t*-test, ANOVA and repeated measures ANOVA as indicated. Post hoc tests were performed using either Sidak’s post hoc analysis or uncorrected Fisher’s LSD as indicated. The criterion for significance for all analyses was *p < 0.05; **p < 0.01; ***p < 0.001. Data are reported as mean ± SEM.

### Supplementary information

Supplementary Material - Methods

supplementary figure legends

Supplementary Figure 1

Supplementary Figure 2

Supplementary Figure 3

Supplementary Figure 4

Supplementary Figure 5

Supplementary Figure 6

Supplementary Figure 7

Supplementary Table 1

## References

[1] Knudsen EI (2004). Sensitive periods in the development of the brain and behavior. J Cogn Neurosci.

[2] Steinberg L. Pubertal maturation and parent-adolescent distance: an evolutionary perspective. 1989. https://psycnet.apa.org/record/1989-97743-003.

[3] Suri D, Teixeira CM, Cagliostro MKC, Mahadevia D, Ansorge MS (2015). Monoamine-sensitive developmental periods impacting adult emotional and cognitive behaviors. Neuropsychopharmacology..

[4] Mejia JM, Ervin FR, Baker GB, Palmour RM (2002). Monoamine oxidase inhibition during brain development induces pathological aggressive behavior in mice. Biol Psychiatry.

[5] Cases O, Seif I, Grimsby J, Gaspar P, Chen K, Pournin S (1995). Aggressive behavior and altered amounts of brain serotonin and norepinephrine in mice lacking MAOA. Science.

[6] Yu Q, Teixeira CM, Mahadevia D, Huang Y, Balsam D, Mann JJ (2014). Dopamine and serotonin signaling during two sensitive developmental periods differentially impact adult aggressive and affective behaviors in mice. Mol Psychiatry.

[7] Buckholtz JW, Meyer-Lindenberg A (2008). MAOA and the neurogenetic architecture of human aggression. Trends Neurosci.

[8] Brunner H, Nelen M, Breakefield X, Ropers H, van Oost B (1993). Abnormal behavior associated with a point mutation in the structural gene for monoamine oxidase A. Science.

[9] Wahlstrom D, Collins P, White T, Luciana M (2010). Developmental changes in dopamine neurotransmission in adolescence: behavioral implications and issues in assessment. Brain Cogn.

[10] Wahlstrom D, White T, Luciana M (2010). Neurobehavioral evidence for changes in dopamine system activity during adolescence. Neurosci Biobehav Rev.

[11] Hughes RN, Bakhurin KI, Petter EA, Watson GDR, Kim N, Friedman AD (2020). Ventral tegmental dopamine neurons control the impulse vector during motivated behavior. Curr Biol.

[12] Hammerslag LR, Waldman AJ, Gulley JM (2014). Effects of amphetamine exposure in adolescence or young adulthood on inhibitory control in adult male and female rats. Behav Brain Res.

[13] Tirelli E, Laviola G, Adriani W (2003). Ontogenesis of behavioral sensitization and conditioned place preference induced by psychostimulants in laboratory rodents. Neurosci Biobehav Rev.

[14] Walker DM, Bell MR, Flores C, Gulley JM, Willing J, Paul MJ (2017). Adolescence and reward: making sense of neural and behavioral changes amid the chaos. J Neurosci.

[15] Reynolds LM, Flores C. Adolescent dopamine development. In: Martin CR, Preedy VR, Rajendram R, Eds. Diagnosis, management and modeling of neurodevelopmental disorders. London: Academic Press, 2021. p. 295–304. 10.1016/b978-0-12-817988-8.00026-9.

[16] Caballero A, Granberg R, Tseng KY (2016). Mechanisms contributing to prefrontal cortex maturation during adolescence. Neurosci Biobehav Rev.

[17] Siever LJ (2008). Neurobiology of aggression and violence. Am J Psychiatry.

[18] Adamantidis AR, Tsai HC, Boutrel B, Zhang F, Stuber GD, Budygin EA (2011). Optogenetic interrogation of dopaminergic modulation of the multiple phases of reward-seeking behavior. J Neurosci.

[19] Izquierdo A, Brigman JL, Radke AK, Rudebeck PH, Holmes A (2017). The neural basis of reversal learning: an updated perspective. Neuroscience..

[20] Yu C, Zhang J, Zuo X, Lian Q, Tu X, Lou C (2021). Correlations of impulsivity and aggressive behaviours among adolescents in Shanghai, China using bioecological model: cross-sectional data from Global Early Adolescent Study. BMJ Open.

[21] Asaoka Y, Won M, Morita T, Ishikawa E, Goto Y (2021). Comparable level of aggression between patients with behavioural addiction and healthy subjects. Transl Psychiatry.

[22] Nautiyal KM, Wall MM, Wang S, Magalong VM, Ahmari SE, Balsam PD (2017). Genetic and modeling approaches reveal distinct components of impulsive behavior. Neuropsychopharmacology..

[23] Wang Q, Chen C, Cai Y, Li S, Zhao X, Zheng L (2016). Dissociated neural substrates underlying impulsive choice and impulsive action. Neuroimage..

[24] Bari A, Robbins TW (2013). Inhibition and impulsivity: behavioral and neural basis of response control. Prog Neurobiol.

[25] McDonald MP, Wong R, Goldstein G, Weintraub B, Cheng SY, Crawley JN (1998). Hyperactivity and learning deficits in transgenic mice bearing a human mutant thyroid hormone β1 receptor gene. Learn Mem.

[26] Niv Y, Joel D, Dayan P (2006). A normative perspective on motivation. Trends Cogn Sci.

[27] Schultz W, Dayan P, Montague PR (1997). A neural substrate of prediction and reward. Science..

[28] Bayer HM, Glimcher PW (2005). Midbrain dopamine neurons encode a quantitative reward prediction error signal. Neuron..

[29] Flagel SB, Clark JJ, Robinson TE, Mayo L, Czuj A, Willuhn I (2010). A selective role for dopamine in stimulus–reward learning. Nature..

[30] Syed ECJ, Grima LL, Magill PJ, Bogacz R, Brown P, Walton ME (2016). Action initiation shapes mesolimbic dopamine encoding of future rewards. Nat Neurosci.

[31] Cunha C, Smiley JF, Chuhma N, Shah R, Bleiwas C, Menezes EC, et al. Perinatal interference with the serotonergic system affects VTA function in the adult via glutamate co-transmission. Mol Psychiatry. 2020. 10.1038/s41380-020-0763-z. 10.1038/s41380-020-0763-z PMC7657958 32398719

[32] Mahadevia D, Saha R, Manganaro A, Chuhma N, Ziolkowski-Blake A, Morgan AA (2021). Dopamine promotes aggression in mice via ventral tegmental area to lateral septum projections. Nat Commun.

[33] Jing MY, Han X, Zhao TY, Wang ZY, Lu GY, Wu N (2019). Re-examining the role of ventral tegmental area dopaminergic neurons in motor activity and reinforcement by chemogenetic and optogenetic manipulation in mice. Metab Brain Dis.

[34] Burns RS, Chiueh CC, Markey SP, Ebert MH, Jacobowitz DM, Kopin IJ (1983). A primate model of parkinsonism: selective destruction of dopaminergic neurons in the pars compacta of the substantia nigra by N-methyl-4-phenyl-1,2,3,6-tetrahydropyridine. Proc Natl Acad Sci USA.

[35] Mirenowicz J, Schultz W (1996). Preferential activation of midbrain dopamine neurons by appetitive rather than aversive stimuli. Nature..

[36] Jin X, Costa RM (2010). Start/stop signals emerge in nigrostriatal circuits during sequence learning. Nature..

[37] Noisin EL, Thomas WE (1988). Ontogeny of dopaminergic function in the rat midbrain tegmentum, corpus striatum and frontal cortex. Brain Res.

[38] Spear LP (2000). The adolescent brain and age-related behavioral manifestations. Neurosci Biobehav Rev.

[39] Kalsbeek A, Voorn P, Buijs RM, Pool CW, Uylings HB (1988). Development of the dopaminergic innervation in the prefrontal cortex of the rat. J Comp Neurol.

[40] Moll GH, Mehnert C, Wicker M, Bock N, Rothenberger A, Rüther E (2000). Age-associated changes in the densities of presynaptic monoamine transporters in different regions of the rat brain from early juvenile life to late adulthood. Brain Res Dev Brain Res.

[41] Teicher MH, Andersen SL, Hostetter JC (1995). Evidence for dopamine receptor pruning between adolescence and adulthood in striatum but not nucleus accumbens. Brain Res Dev Brain Res.

[42] Lidow MS, Rakic P (1992). Scheduling of monoaminergic neurotransmitter receptor expression in the primate neocortex during postnatal development. Cereb Cortex.

[43] Tarazi FI, Baldessarini RJ (2000). Comparative postnatal development of dopamine D(1), D(2) and D(4) receptors in rat forebrain. Int J Dev Neurosci.

[44] McCutcheon JE, Marinelli M (2009). Age matters. Eur J Neurosci.

[45] Stamford JA (1989). Development and ageing of the rat nigrostriatal dopamine system studied with fast cyclic voltammetry. J Neurochem.

[46] Reynolds LM, Yetnikoff L, Pokinko M, Wodzinski M, Epelbaum JG, Lambert LC (2019). Early adolescence is a critical period for the maturation of inhibitory behavior. Cereb Cortex.

[47] Putzier I, Kullmann PHM, Horn JP, Levitan ES (2009). Dopamine neuron responses depend exponentially on pacemaker interval. J Neurophysiol.

[48] Khaliq ZM, Bean BP (2010). Pacemaking in dopaminergic ventral tegmental area neurons: depolarizing drive from background and voltage-dependent sodium conductances. J Neurosci.

[49] Tossell K, Dodhia RA, Galet B, Tkachuk O, Ungless MA (2021). Tonic GABAergic inhibition, via GABAA receptors containing αβƐ subunits, regulates excitability of ventral tegmental area dopamine neurons. Eur J Neurosci.

[50] Mao D, Gallagher K, McGehee DS (2011). Nicotine potentiation of excitatory inputs to ventral tegmental area dopamine neurons. J Neurosci.

[51] Overton PG, Clark D (1997). Burst firing in midbrain dopaminergic neurons. Brain Res Brain Res Rev.

[52] Johnson SW, Seutin V, North RA (1992). Burst firing in dopamine neurons induced by N-methyl-D-aspartate: role of electrogenic sodium pump. Science..

[53] Lohani S, Martig AK, Underhill SM, DeFrancesco A, Roberts MJ, Rinaman L (2018). Burst activation of dopamine neurons produces prolonged post-burst availability of actively released dopamine. Neuropsychopharmacology.

[54] Moore H, Rose HJ, Grace AA (2001). Chronic cold stress reduces the spontaneous activity of ventral tegmental dopamine neurons. Neuropsychopharmacology.

[55] Chang CH, Grace AA (2013). Amygdala β-noradrenergic receptors modulate delayed downregulation of dopamine activity following restraint. J Neurosci.

[56] Zimmerman EC, Grace AA (2016). The nucleus reuniens of the midline thalamus gates prefrontal-hippocampal modulation of ventral tegmental area dopamine neuron activity. J Neurosci.

[57] Terranova ML, Laviola G, de Acetis L, Alleva E (1998). A description of the ontogeny of mouse agonistic behavior. J Comp Psychol.

[58] Manduca A, Servadio M, Damsteegt R, Campolongo P, Vanderschuren LJ, Trezza V (2016). Dopaminergic neurotransmission in the nucleus accumbens modulates social play behavior in rats. Neuropsychopharmacology.

[59] Panksepp J (1981). The ontogeny of play in rats. Dev Psychobiol.

[60] Urošević S, Collins P, Muetzel R, Lim K, Luciana M (2012). Longitudinal changes in behavioral approach system sensitivity and brain structures involved in reward processing during adolescence. Dev Psychol.

[61] Doremus-Fitzwater TL, Spear LP (2016). Reward-centricity and attenuated aversions: an adolescent phenotype emerging from studies in laboratory animals. Neurosci Biobehav Rev.

[62] Smith LK, Field EF, Forgie ML, Pellis SM (1996). Dominance and age-related changes in the play fighting of intact and post-weaning castrated male rats (Rattus norvegicus). Aggress Behav.

[63] Anderson ER, Bell NJ, Fischer JL, Munsch J, Peek CW, Sorell GT. Applying a risk-taking perspective. In: Bell NJ, Bell RW Eds. Adolescent risk taking. Newbury Park, CA: Sage Publications; 1993. 165–85.

[64] Adriani W, Chiarotti F, Laviola G (1998). Elevated novelty seeking and peculiar d-amphetamine sensitization in periadolescent mice compared with adult mice. Behav Neurosci.

[65] Burton CL, Fletcher PJ (2012). Age and sex differences in impulsive action in rats: the role of dopamine and glutamate. Behav Brain Res.

[66] Doremus-Fitzwater TL, Spear LP (2011). Amphetamine-induced incentive sensitization of sign-tracking behavior in adolescent and adult female rats. Behav Neurosci.

[67] Fuhrmann D, Knoll LJ, Blakemore SJ (2015). Adolescence as a sensitive period of brain development. Trends Cogn Sci.

[68] Kopec AM, Smith CJ, Ayre NR, Sweat SC, Bilbo SD (2018). Microglial dopamine receptor elimination defines sex-specific nucleus accumbens development and social behavior in adolescent rats. Nat Commun.

[69] Nelson RJ, Trainor BC (2007). Neural mechanisms of aggression. Nat Rev Neurosci.

[70] Heinz AJ, Beck A, Meyer-Lindenberg A, Sterzer P, Heinz A (2011). Cognitive and neurobiological mechanisms of alcohol-related aggression. Nat Rev Neurosci.

[71] Coccaro EF, Sripada CS, Yanowitch RN, Phan KL (2011). Corticolimbic function in impulsive aggressive behavior. Biol Psychiatry.

[72] El-Ghundi M, O’Dowd BF, George SR (2007). Insights into the role of dopamine receptor systems in learning and memory. Rev Neurosci.

[73] Keiflin R, Janak PH (2015). Dopamine prediction errors in reward learning and addiction: from theory to neural circuitry. Neuron..

[74] Garrett DD, Nagel IE, Preuschhof C, Burzynska AZ, Marchner J, Wiegert S (2015). Amphetamine modulates brain signal variability and working memory in younger and older adults. Proc Natl Acad Sci USA.

[75] Lapish CC, Balaguer-Ballester E, Seamans JK, Phillips AG, Durstewitz D (2015). Amphetamine exerts dose-dependent changes in prefrontal cortex attractor dynamics during working memory. J Neurosci.

[76] Weiner I, Feldon J (1986). Reversal and nonreversal shifts under amphetamine. Psychopharmacology.

[77] Sesia T, Bulthuis V, Tan S, Lim LW, Vlamings R, Blokland A (2010). Deep brain stimulation of the nucleus accumbens shell increases impulsive behavior and tissue levels of dopamine and serotonin. Exp Neurol.

[78] Pattij T, Janssen MCW, Vanderschuren LJMJ, Schoffelmeer ANM, van Gaalen MM (2007). Involvement of dopamine D1 and D2 receptors in the nucleus accumbens core and shell in inhibitory response control. Psychopharmacology.

[79] Golden SA, Jin M, Heins C, Venniro M, Michaelides M, Shaham Y (2019). Nucleus accumbens Drd1-expressing neurons control aggression self-administration and aggression seeking in mice. J Neurosci.

[80] Flores-Dourojeanni JP, van Rijt C, van den Munkhof MH, Boekhoudt L, Luijendijk MCM, Vanderschuren LJMJ (2021). Temporally specific roles of ventral tegmental area projections to the nucleus accumbens and prefrontal cortex in attention and impulse control. J Neurosci.

[81] Li B, Nguyen TP, Ma C, Dan Y (2020). Inhibition of impulsive action by projection-defined prefrontal pyramidal neurons. Proc Natl Acad Sci USA.

[82] Friedman NP, Robbins TW (2022). The role of prefrontal cortex in cognitive control and executive function. Neuropsychopharmacology..

[83] Reynolds LM, Pokinko M, Torres-Berrío A, Cuesta S, Lambert LC, Del Cid Pellitero E (2018). DCC receptors drive prefrontal cortex maturation by determining dopamine axon targeting in adolescence. Biol Psychiatry.

[84] Barratt ES, Felthous AR (2003). Impulsive versus premeditated aggression: implications for mens rea decisions. Behav Sci Law.

[85] Gollan JK, Lee R, Coccaro EF (2005). Developmental psychopathology and neurobiology of aggression. Dev Psychopathol.

[86] Meloy JR (2006). Empirical basis and forensic application of affective and predatory violence. Aust N Z J Psychiatry.

[87] Lipari RN, Park-Lee E. Key substance use and mental health indicators in the United States: results from the 2018 National Survey on Drug Use and Health. Substance Abuse and Mental Health Services Administration; 2019. 29431966

[88] Briars L, Todd T (2016). A review of pharmacological management of attention-deficit/hyperactivity disorder. J Pediatr Pharmacol Ther.

[89] Wilens TE, Spencer TJ (2010). Understanding attention-deficit/hyperactivity disorder from childhood to adulthood. Postgrad Med.

[90] Lakhan SE, Kirchgessner A (2012). Prescription stimulants in individuals with and without attention deficit hyperactivity disorder: misuse, cognitive impact, and adverse effects. Brain Behav.

[91] Schifano F, Napoletano F, Chiappini S, Guirguis A, Corkery JM, Bonaccorso S (2021). New/emerging psychoactive substances and associated psychopathological consequences—Corrigendum. Psychol Med.

[92] Faraone SV, Rostain AL, Montano CB, Mason O, Antshel KM, Newcorn JH (2020). Systematic review: Nonmedical use of prescription stimulants: risk factors, outcomes, and risk reduction strategies. J Am Acad Child Adolesc Psychiatry.

[93] Demireva EY, Suri D, Morelli E, Mahadevia D, Chuhma N, Teixeira CM (2020). 5-HT2C receptor blockade reverses SSRI-associated basal ganglia dysfunction and potentiates therapeutic efficacy. Mol Psychiatry.

[94] Morelli E, Moore H, Rebello TJ, Gray N, Steele K, Esposito E (2011). Chronic 5-HT transporter blockade reduces DA signaling to elicit basal ganglia dysfunction. J Neurosci.

