# Supplementary material for: Dopamine transporter blockade during adolescence increases adult dopamine function, impulsivity, and aggression
Source: Mol Psychiatry. 2023 Aug 2;28(8):3512–23. doi: 10.1038/s41380-023-02194-w (PMC10618097; doi:10.1038/s41380-023-02194-w)
Supplement: Supplementary file 1 — Supplementary Material - Methods [file 41380_2023_2194_MOESM1_ESM.docx]

**Supplementary Methods**

**Subjects.**

Mice (129SvEv/Tac) were bred at Columbia Psychiatry, New York State Psychiatric Institute. Mice used for experiments were born to litters containing 4 - 6 pups. Mice were separated by sex and weaned into groups of five mice per cage at P26. Only male mice were used to examine aggression behavior and amphetamine response. Male and female mice were used to assess operant behavior, working memory, and electrophysiology. No interactions between sex other independent variable were detected, and data were collapsed for sex as a consequence. Mice were treated once daily during the peri-adolescence period of postnatal day 32-41 (P32-41) with VEH or GBR. For the amphetamine challenge and aggression experiments, additional groups were treated from P22-31 and P42-51 with VEH or GBR. Treatment consisted of intraperitoneal (i.p.) drug injections of vehicle (VEH, 0.9% NaCl, 5 ml/kg) or the DAT blocker GBR12909 (GBR, 20 mg/kg). Injections were administered daily between 10 and 12 am. To selectively express channelrhodopsin 2 (ChR2) in DAergic neurons, we crossed a DA-transporter Cre-driver-line (Dat^IRES-Cre^) [^1^](https://paperpile.com/c/kTA7jh/2RpcZ) with the ROSA26-floxed-STOP-CAG-ChR2-EYFP (ChR2^fl/fl^) Ai32 line [^2^](https://paperpile.com/c/kTA7jh/OcwOv). We used Dat^IRES-Cre/+^;ChR2^fl/fl^ (DatCre;ai32, experimental) and Dat^+/+^;ChR2^fl/fl^ (Wt;ai32, control). Animals were maintained on a 12-hour light-dark cycle (lights on at 7:00 am) and provided with food and water *ad libitum*. Animal testing was conducted in accordance with the *Principles of Laboratory Animal Care* National Institute of Health (NIH) guidelines and the institutional animal committee guidelines.

**Behaviors.**

**Amphetamine induced locomotor activity**

Locomotor activity was assessed using the open field test. Mice were allowed to explore brightly lit (800-900 lux) plexiglass activity chambers (43.2 × 43.2 × 30.5 cm, length × width × height; model ENV-520; Med Associates, Georgia, VT) equipped with infrared beams located 1.5cm above the chamber floor. Beams were positioned such that both horizontal and vertical activity was detectable. Mice were placed into the center of the open field and activity was recorded for 30min to assess baseline locomotion. To assess amphetamine-induced locomotion, the system was programmed to halt recording 30 min after starting a session, at which point mice were injected with amphetamine (0.5 mg/kg, i.p.) or saline (SAL, 0.9% NaCl) and placed back in the open field. Typical amphetamine doses used to observe effects on locomotor activity range from 0.5 to 3 mg/kg [^3–5^](https://paperpile.com/c/kTA7jh/irv8+ES2W+Ocl3). We chose 0.5 mg/kg because it provides a dynamic range that allows us to detect augmented and blunted responses in 129SvEv/Tac mice. Higher doses (5-10 mg/kg) of amphetamine can lead to stereotyped behaviors and ceiling locomotor effects [^6^](https://paperpile.com/c/kTA7jh/43bs). Recording of locomotor activity was resumed for another 60 min immediately after injections. The total distance traveled over the 90 mins was measured and binned every 5 minutes.

**Isolation-induced aggression test**

In order to assess aggressive behavior, the isolation-induced aggression paradigm was used [^7^](https://paperpile.com/c/kTA7jh/FYIJL). The home cage was divided in half by a perforated partition made of clear plastic. A pair of mice with the same treatment was placed in the cage, one in each compartment. The mice were able to see, hear and smell each other through the holes in the plastic divider, but physical interaction was blocked. Mice were housed in this condition for 10 days before the test day. On the test day, dividers were taken out and mice were allowed to freely interact for 15 minutes. The time spent fighting was scored as a sum of the time spent biting, tail rattling, and mounting. Given that behavioral data were collected across more than one cohort, all data were normalized to the respective VEH control mice. All fights were video recorded to allow for any additional post-hoc analysis.

**T-maze**

Food-restricted mice were placed in the start arm of a T-maze and trained in the forced run to retrieve a reward from an arm by closing the opposite arm. During trial sessions, also called choice run, animals were placed in the start arm and given the choice to choose between the two arms, however, the reinforcement was only present in the arm not previously entered during the forced run of that trial. We tested different delays after the forced run: 10, 30, 45, and 60 seconds.

**Operant behaviors**

Operant Conditioning

Studies were conducted in 4 identical operant chambers (7 × 6 × 7.25) inch with stainless steel grid flooring illuminated by a house light (Med Associates Inc., St Albans, VT), individually enclosed in sound-insulating, ventilated cubicles. Each chamber had two ultra-sensitive retractable stainless-steel levers placed 0.89 inches above the chamber floor situated on either side of a receptacle. The receptacle was equipped with head entry detection for delivery of liquid reward (0.01cc evaporated milk Nestle, Elizabeth, NJ) to mice. A Dell computer equipped with MED-PC IV (Med Associates Inc., St Albans, VT) computer software delivered stimuli and collected behavioral data. Operant training and testing were run 7 days a week. Mice were maintained at ~85% of their free-feeding weight by providing *ad libitum* access to chow for 1.5 h following each day’s operant conditioning session during the light cycle. Water was provided *ad libitum* throughout the experiment.

Dipper training

For the first phase of training, animals learned to retrieve the milk reward by making head entries into the receptacle. Mice were placed inside the chambers with the dipper in the raised position, providing access to a drop of evaporated milk. The dipper was retracted 10 s after the first head entry into the feeder trough. A variable intertrial interval (average 40s) ensued, followed by a new trial identical to the first. The session ended after 30 min or 20 dipper presentations. On the following day, mice underwent another session similar to the first, except that the dipper retraction was response-independent. During each trial, the dipper was raised for 8 s and then lowered independently of whether mice had made a head entry. The session ended after 30 min or 20 dipper presentations. Following 4 days of training, all mice met the criterion of retrieving at least 90% of the 30 rewards presented, within 8 s of their presentation.

Continuous reinforcement training (CRF).

In the subsequent phase of training, mice were required to press a lever to earn the reward. An evaporated milk was dabbed on the lever during the initial sessions to facilitate interaction with the lever. At the start of each trial, the lever was extended into the chamber, and every lever press was reinforced with a reward. The lever was retracted after every two times the mouse earned a reward and then was re-extended after a variable inter-trial interval, averaging 30 s. The session ended when the mouse earned 60 reinforcements or 1 h elapsed. Mice continued to undergo daily continuous reinforcement sessions until they earned 50 out of 60 rewards in three consecutive sessions. Each mouse was trained on a randomly assigned lever (left or right), which remained consistent for the go/no-go, progressive ratio (PR), behavioral flexibility and delayed discounting paradigms.

Stimulus based learning and learning flexibility

Mice were trained to discriminate between two different visual stimuli, a steady light (S^+^) during which a lever press response resulted in a reward and a flashing light (S^-^) during which the lever press response did not earn a reward. To test cognitive flexibility, we then switched the contingencies of the S^+^ and S^-^ stimuli such the steady light was associated with no rewards (S^-^) and the flashing light was associated with rewards (S^+^).

Progressive ratio

Prior to the commencement of the Progressive ratio test the animals were trained on random ratio (RR) training. In the RR schedule training, the lever remained extended throughout the session, however, an average number of lever press responses were required to obtain a single reinforcement. The average number of responses required to obtain one reinforcement was defined by the RR schedule. All mice began on the RR 5 schedule, meaning that a lever press was reinforced following an average of 5 lever press responses. When a mouse earned at least 40 rewards in one session, the RR schedule was increased. The RR schedules used were 5, 10, and 20. When all mice reached the criterion of 40 rewards in one session on the RR 20 schedule, they began experimental testing on a progressive ratio schedule. Mice continued to undergo RR 5, 10, and 20 sessions between each progressive ratio session to reinstate the lever press behavior.

Following RR training, mice were run on a PR schedule to assess motivation behavior. In the PR there was a progressive increase in the number of lever presses required to obtain every subsequent reinforcer. The increase in the number of lever presses was dictated by the PR schedule. The mice were tested on three schedules of PR namely, PR+2, PR+5, and PRx2. In PR+2 and PR+5 the number of lever presses required to get every subsequent reinforcer was enhanced by 2 and 5 respectively. On the PRx2 schedule, the number of lever presses required to receive a reward doubled following each reward. The session ended following either 2 h or a 3 min period in which no lever presses were recorded [^8^](https://paperpile.com/c/kTA7jh/peCNl). The total number of lever presses summed over the session, and the session time was analyzed to assess the motivation of the animal. The total number of lever presses rather than break point was analyzed to provide a continuous rather than categorical variable.

Delayed discounting

In the Delayed Discounting paradigm, we assessed the tolerance to reward-associated delay. Animals were given access to 2 levers, a small-immediate reward lever, and a larger-delayed reward lever. The large reward-associated delay was gradually increased across testing days from 0-10s. Testing began with forced trials on each lever to reinforce lever-associated delay and reward magnitude, followed by free choice trials where the animals were required to choose between the small immediate versus large-delayed reward. Total of 27 trials 3 and 3 forced trials on each lever and 21 free-choice trials. In the choice trials, both levers are out and the animal needs to respond to one of them. As soon as the animal makes a response the levers withdraw and the animal receives one or three dippers depending on their choice. In the forced trials, only one of the levers is out and it stays out till the animal makes a response to it. This is predominantly to inform the animal about the reward contingency associated with each lever. Once 75% of the responses were made on the large reward lever the animals were moved to the test. The animals are tested on 6 delay criteria- 0s, 2s, 4s, 6s, 8s, and 10s. Each criterion is tested for 3 days- each day has 6 forced trials (3 on each lever) and 21 choice trials.

Go/No-Go

Mice were first trained on Go-only trials in which they were required to press a lever within 5 s of its presentation to receive a reward. If the 5 s elapsed with no response, the lever would retract with no reward presentation, and a new ITI (average 40 s) would begin. House light was kept on continuously through the trials and the ITIs. Mice were trained on these 5 s Go-only trials until they earned at least 50 rewards from 60 trials for at least three consecutive sessions. Once this criterion was achieved, the testing phase commenced. The Go/NoGo testing phase consisted of 30 go trials pseudo-randomly interspersed with an equal number of NoGo trials. During the No-Go trials, the lever was presented simultaneously with a NoGo cue (the house lights turning off, and a small LED lever light turning on). The lack of any lever press for the 5 s duration of the NoGo trial, resulted in a reward. A lever press during this period caused the lever to retract, the house lights to turn on, the LED light to turn off, and a new ITI to begin without any reward presentation. Mice were run for 12 days on the 5s Go/NoGo test, and the number of incorrect No-Go trials and number of correct Go trials were analyzed. From day 13 mice were trained for 3 days on the same task with 10s duration of Go and NoGo trials. These test days were identical to the 5 s Go/NoGo testing phase except that the animals were required to refrain from pressing the lever for a period of 10s during the NoGo trials. The duration and nature of the Go trials remained unchanged.

For optical stimulation protocols, we started cable training once the animals reached > 30 “dippers” and > 30 “HeadIns” per session in the CRF task. The optogenetic stimulation started when animals reached at least 40% correct of the NoGo trials for 3 consecutive days. Photostimulation epochs at 473 nm, 20 Hz, 10 ms, and 10 mW lasted 3 seconds (1 second before the trial and 2 seconds into the trial) for 50% of the trials in a counterbalanced manner from day 9 to 14 in the VTA experiment and on day 9 for the SNc experiment.

**Open field**

The open field apparatus consisted of square Plexiglas activity chambers equipped with lateral infrared detectors to track horizontal and vertical animal activity. Mice were allowed to walk freely over a 12-min trial. Optogenetic stimulation was delivered in 3-min OFF, 3-min ON cycles. The total distance traveled was analyzed.

**Slice electrophysiology**

P61-80 male mice were used for recording from ventral midbrain dopamine neurons. 300 µm horizontal slices of ventral midbrain were prepared as described before[^9^](https://paperpile.com/c/kTA7jh/3kj4C) . The slices were mounted in a recording chamber on an upright microscope (BX61WI, Olympus) continuously perfused with artificial cerebrospinal fluid (ACSF; in mM 125 NaCl, 2.5 KCl, 25 NaHCO_3_, 1.25 NaH_2_PO_4_, 2 CaCl_2_, 1 MgCl_2_ and 25 glucose, pH 7.4) saturated with 95%O_2_-5%CO_2_. The ACSF in the chamber was maintained at 31 - 33 ºC (TC 344B Temperature Controller, Warner Instruments). Whole-cell recordings were made from visually identified DA neurons. The VTA was recognized as the medial side of the optic tract, and recording was done from large cells in the lateral VTA. Current clamp recording was done using an Axopatch 200B amplifier (Molecular Devices) in fast current-clamp mode. Recording pipettes (3-6 MΩ) were filled with a K^+^-methanesulfonate based solution (in mM: 135 KMeSO_4_, 5 KCl, 2 MgCl_2_, 0.1 CaCl_2_, 10 HEPES, 1 EGTA, 2 ATP-Na_2_, 0.1 GTP, pH 7.25). Series resistance (5-16 MΩ) was not compensated. Liquid junction potential (~ 8 mV) was adjusted online. For measuring membrane properties, 700 ms duration -400 to +300 pA (50 pA step) currents were injected. 0.2% Alexa 594 was included in the pipette solution for post-recording identification of cells. Data were filtered at 5 kHz using a 4-pole Bessel filter and digitized (ITC-18 interface, ALA Scientific Instruments) at 100 µsec intervals. Data acquisition and analysis were done with Axograph X (Axograph Scientific) or pClamp 10 (Molecular Devices). Firing frequency was measured just after the whole-cell with automatic spike detection function (threshold = 0 mV). Since DA neurons fired continuously and it was difficult to identify the location of the resting-state, resting membrane potential was measured as the averaged potential of traces after action potential truncated. Input impedance was measured from -100 pA current injection traces. Action potential threshold was measured as the point where the change of membrane potential reached 10 mV/ms. Action potential amplitude and after-hyperpolarization potentials were measured as positive and negative peak potentials, respectively, from resting membrane potential. DA neuron identity was confirmed in recorded slices. Slices were fixed with 4% PFA in PBS overnight at 4ºC then washed with PBS and kept in PBS with 0.1% sodium azide at 4 ºC for tyrosine hydroxylase (TH) immunostaining. Brain sections were washed in 0.1 M phosphate buffer (PB) for 15 min on a rocker at room temperature (RT). Sections were blocked in 10% Horse Serum, 0.01% Triton X 100 in 0.1 M PB for 4 - 6 hours at RT on a shaker. We then incubated in rabbit anti-TH primary antibody (1:800; catalog no. AB152, Millipore Sigma, Darmstadt, Germany) in 0.1 M PB for 3 nights at 4º C on a shaker. After washes in 0.1 M PB we incubated in a donkey anti-rabbit secondary antibody (1:200; catalog no. 711-545-152, Jackson Immunoresearch 488) for 3 hours at RT. Sections were washed 3 X in 0.1 M PB, mounted on Superfrost slides (Fisher Scientific; catalog no.12-550-15) and cover slipped with Prolong Gold Antifade (catalog no. P36930; Invitrogen). Sections were visualized on a laser confocal scanning microscope (Leica, TCS SP8).

**In vivo recordings**

Extracellular recording microelectrodes (2.0 mm OD borosilicate glass capillary tubing, ~1µm tip diameter, impedance 5 – 10 MOhms) were filled with 2 M NaCl containing 2% pontamine sky blue dye. The electrode signal was amplified at 1000x gain with a 2-channel microelectrode AC amplifier (A-M systems), and filtered using a 10 - 100 kHz band pass filter. Mice were anesthetized with 400 mg/kg Chloral hydrate for induction and 100 mg/kg Chloral Hydrate for maintenance. The body temperature was monitored by a rectal probe and maintained between 36 and 37º C using a water circulation pad placed under a clean gauze pad. A hole was drilled through the skull overlying the brain structures of interest, and the dura was then punctured. A glass electrode was lowered into the VTA (anterior/posterior (AP) -3.5 mm, medial/lateral (ML) -0.5 mm, and dorsal/ventral (DV) -3.5mm), and SNc (AP -3.5, ML -1.5, DV −5.5). DAergic and non-DAergic neurons were identified using established electrophysiological criteria. In brief, neurons exhibit slow-frequency firing rates (0.5 – 10 Hz) and irregular firing pattern interspersed with high-frequency burst events as well as triphasic waveforms of long duration (>1.1 ms from beginning to trough)[10–12](https://paperpile.com/c/kTA7jh/cjwu7+hhg56+j9jtZ). Once isolated, spontaneous activities were recorded for 3 min. The DAergic activity in the VTA and SNc was systematically investigated by recording from 4 - 6 tracks within this region per mouse. Three parameters of activity were sampled: (a) the number of spontaneously active DAergic neurons recorded per electrode track (i.e., population activity), (b) basal firing rate (average number of spikes/second per cell), and (c) bursting activity. The start of a burst was defined by the occurrence of two spikes with an inter-spike interval < 80 ms, and its termination by the occurrence of an inter-spike interval > 160 ms as previously described [^10^](https://paperpile.com/c/kTA7jh/cjwu7). Data were continuously digitized with a Neurocorder DR-890 A/D converter (Cygnus Technologies, Delaware Water Gap, PA) and stored on a hard drive using a data acquisition board (Microstar Labs, Bellevue, WA) interfaced with a Microsoft Windows-based Pentium PC. Analysis was performed with custom-designed software (Neuroscope©). Data were averaged per animal.

**Fiber photometry data analysis**

The 405 nm control trace was fitted to and subtracted from the 470 nm trace to calculate a dF, which was then divided by the fitted control trace to calculate a dF/F. The dF/F traces were converted to z-scores based on their median absolute deviation from the median and aligned to the lever extensions. The traces were then normalized to the 5 second window baseline prior to the lever extension and dF/F was quantified over 10 seconds after lever extension for correct and incorrect Go/No-go trials. Data are presented as the average of trials for all phases of the different days of the Go/No-Go task as well as separated into the early, middle, and late phases. For the asymmetric design, boundaries were defined by NoGo performance with < 20% for the early phase, between 20% and 40% for the middle phase, and > 40% for the late phase. This definition happened to parse the 30 days of assessment into 10 days each (early phase: days 1-10, middle phase: days 11-20, and late phase: days 21-30). For the symmetric design, mice were run for 15 days and the three phases consisted of 5 days each (early phase: days 1-5, middle phase: days 6-10, and late phase: days 11-15). The anticipatory peak amplitude was quantified by subtracting the initial dF/F value at lever extension from the maximum dF/F peak within 0.7 - 1.4 sec for dataset 1 and 0 - 1 sec for dataset 2.

1. [Bäckman, C. M. *et al.* Characterization of a mouse strain expressing Cre recombinase from the 3’ untranslated region of the dopamine transporter locus. *Genesis* **44**, 383–390 (2006).](http://paperpile.com/b/kTA7jh/2RpcZ)

2. [Madisen, L. *et al.* A toolbox of Cre-dependent optogenetic transgenic mice for light-induced activation and silencing. *Nat. Neurosci.* **15**, 793–802 (2012).](http://paperpile.com/b/kTA7jh/OcwOv)

3. [Yates, J. W., Meij, J. T. A., Sullivan, J. R., Richtand, N. M. & Yu, L. Bimodal effect of amphetamine on motor behaviors in C57BL/6 mice. *Neurosci. Lett.* **427**, 66–70 (2007).](http://paperpile.com/b/kTA7jh/irv8)

4. [Salahpour, A. *et al.* Increased amphetamine-induced hyperactivity and reward in mice overexpressing the dopamine transporter. *Proc. Natl. Acad. Sci. U. S. A.* **105**, 4405–4410 (2008).](http://paperpile.com/b/kTA7jh/ES2W)

5. [Yu, Q. *et al.* Dopamine and serotonin signaling during two sensitive developmental periods differentially impact adult aggressive and affective behaviors in mice. *Mol. Psychiatry* **19**, 688–698 (2014).](http://paperpile.com/b/kTA7jh/Ocl3)

6. [McNamara, R. K. *et al.* Dose-response analysis of locomotor activity and stereotypy in dopamine D3 receptor mutant mice following acute amphetamine. *Synapse* **60**, 399–405 (2006).](http://paperpile.com/b/kTA7jh/43bs)

7. [Takahashi, A., Nagayasu, K., Nishitani, N., Kaneko, S. & Koide, T. Control of intermale aggression by medial prefrontal cortex activation in the mouse. *PLoS One* **9**, e94657 (2014).](http://paperpile.com/b/kTA7jh/FYIJL)

8. [Nautiyal, K. M. *et al.* Genetic and Modeling Approaches Reveal Distinct Components of Impulsive Behavior. *Neuropsychopharmacology* **42**, 1182–1191 (2017).](http://paperpile.com/b/kTA7jh/peCNl)

9. [Chuhma, N., Tanaka, K. F., Hen, R. & Rayport, S. Functional connectome of the striatal medium spiny neuron. *J. Neurosci.* **31**, 1183–1192 (2011).](http://paperpile.com/b/kTA7jh/3kj4C)

10. [Grace, A. A. & Bunney, B. S. The control of firing pattern in nigral dopamine neurons: burst firing. *J. Neurosci.* **4**, 2877–2890 (1984).](http://paperpile.com/b/kTA7jh/cjwu7)

11. [Grace, A. A. & Bunney, B. S. Nigral dopamine neurons: intracellular recording and identification with L-dopa injection and histofluorescence. *Science* **210**, 654–656 (1980).](http://paperpile.com/b/kTA7jh/hhg56)

12. [Ungless, M. A., Magill, P. J. & Bolam, J. P. Uniform inhibition of dopamine neurons in the ventral tegmental area by aversive stimuli. *Science* **303**, 2040–2042 (2004).](http://paperpile.com/b/kTA7jh/j9jtZ)
