# Supplementary material for: Dopamine transporter blockade during adolescence increases adult dopamine function, impulsivity, and aggression
Source: Mol Psychiatry. 2023 Aug 2;28(8):3512–23. doi: 10.1038/s41380-023-02194-w (PMC10618097; doi:10.1038/s41380-023-02194-w)
Supplement: Supplementary file 2 — supplementary figure legends [file 41380_2023_2194_MOESM2_ESM.docx]

**Supplementary figures**

**Supplementary Figure 1. DAT blockade from P32-41, but not before or after, increases total fighting time. (A)** No change in normalized fight time after P22-31 GBR treatment (N = 9 VEH, N = 9 GBR). **(B)** Increased in normalized fight time after P32-41 GBR treatment (N = 10 VEH, N = 10 GBR). **(C)** No change in normalized fight time after P42-51 GBR treatment (N = 20 VEH, N = 19 GBR). **(D, G)** No change in non-normalized fight time after P22-31 GBR treatment. **(E, H)** Increased fight time after P32-41 GBR treatment. **(F, I)** No change in non-normalized fight time after P42-51 GBR treatment. **(J)** No change in the latency to fight after P22-31 GBR treatment**. (K)** Decreased latency to fight after P32-41 GBR treatment. **(L)** No change in the latency to fight after P42-51 GBR treatment. **(M)** No change in the average bout frequency (number of bouts per 10 min session) of biting, rattling, and mounting after P22-31 GBR treatment**. (N)** Increased average bout frequency of biting, rattling, and mounting after P32-41 GBR treatment. **(O)** Decreased average bout frequency of biting, rattling, and mounting after P42-51 GBR treatment. *p < 0.05, # p < 0.1.

**Supplementary Figure 2. Peri-adolescent GBR treatment increases in vivo DA neuron cell activities.** P32-41 GBR administration increases the number of active cells per track **(A)** as well as the percent of spikes occurring in bursts (**C**). P32-41 GBR administration does not alter overall firing rates **(B)** or burst firing rates **(D)** (N = 34 VEH_VTA_, N = 17 VEH_SNc_, N = 110 GBR_VTA_, N = 47 GBR_SNc_, ). *p < 0.05.

**Supplementary Figure 3**. **Peri-adolescent GBR treatment does not alter the latency to lever press or to retrieve the reward in Go-trials.** In trials that are rewarded by pressing a lever (Go-trials), P32-41 GBR administration does not affect the latency to lever press **(A)** or the latency to retrieve the reward **(B)**. (N = 19 VEH and N = 21 GBR).

**Supplementary Figure 4. VTA dopamine neuronal activity during the Go-NoGo task over time. (A)** Normalized dopamine activity (df/F) aligned to trial start (lever presentation) during the Go-NoGo task, separated for correct Go (cyan) and correct NoGo (magenta) trials during the early learning phase of the task. The first dopamine peak corresponds to the anticipatory peak followed by the consummatory peak, as indicated by arrows. Go trials had no delay between lever press and dipper out, while NoGo trials had 5 s delay. **(B)** Normalized dopamine activity (df/F) during the middle learning phase of the Go-NoGo task. **(C)** Normalized dopamine activity (df/F) during the late learning phases of the Go-NoGo task. Anticipatory DA peak averages for middle learning phase of Correct Go trials are larger than the average anticipatory DA peaks of the corresponding Correct NoGo trials (**D - F**). **(G - I)** Normalized dopamine activity (df/F) aligned to trial start (lever presentation) during the Go-NoGo task, with 4 s delays between lever press and dipper out for both Go and NoGo trials. Anticipatory DA peak averages for early (**J**), middle (**K**), and late (**L**) of Correct Go trials are larger than the average anticipatory DA peaks of the corresponding Correct NoGo trials. **p < 0.01, ***p < 0.001.

**Supplementary Figure 5. *In vivo* optogenetic stimulation of VTA DA neurons increases activity in the open field. (A)** 473 nm light administration into the VTA increased locomotor activity in the open field in DatCre;Ai32 animals but not in WT;Ai32 control animals (N = 12 WT;Ai32, N = 34 DatCre;Ai32). *p < 0.05, ***p < 0.001.

**Supplementary Figure 6**. ***In vivo* optogenetic stimulation of VTA DA neurons does not alter the latency to lever press or to retrieve the reward in Go-trials.** In trials that are rewarded by pressing a lever (Go-trials), optogenetic stimulation of VTA DAergic neurons does not affect the latency to lever press **(A)** or the latency to retrieve the reward **(B)**. (N = 6 WT;Ai32 and N = 7 DatCre;Ai32).

**Supplementary Figure 7**. **Histological validation of fiber optic implant placements.** **(A)** Brain atlas schematic depicting fiber optic ferrule placements in the VTA (blue region with white stars, N = 13) and SNc (grey region with red stars, N = 6) for optogenetic stimulation experiments. **(B)** Brain atlas schematic depicting fiber optic ferrule placement in the VTA (blue region with white stars, N = 1) for fiber photometry recording experiments. (**C**) Exemplary image of fiber optic implant placement (star) and viral GCaMP6s expression in the VTA of a Dat^IRESCre^ mouse, immunostained against GCaMP6s (green), tyrosine hydroxylase (TH, red) and cellular nuclei (DAPI, blue). Of note, this mouse underwent the identical surgery as the mice used for FP recordings in the Go-Nogo test but was tested in a different behavior. Scalebar = 200 μm.

**Supplementary Table 1**. **Statistics for Figure 1.**

**Supplementary Table 2**. **Statistics for Figure 2.**

**Supplementary Table 3**. **Statistics for Figure 3.**

**Supplementary Table 4**. **Statistics for Figure 5.**

**Supplementary Table 5**. **Statistics for Figure 6.**
