# Supplementary material for: Dopamine transporter blockade during adolescence increases adult dopamine function, impulsivity, and aggression
Source: Mol Psychiatry. 2023 Aug 2;28(8):3512–23. doi: 10.1038/s41380-023-02194-w (PMC10618097; doi:10.1038/s41380-023-02194-w)
Supplement: Supplementary file 10 — Supplementary Table 1 [file 41380_2023_2194_MOESM10_ESM.docx]

**Table 1. Statistics for Supplementary Figure 1**

| **Panel** | **Groups comparison** | **Test result** | **P value** | **VEH** | **GBR** | **Norm. VEH** | **Norm. GBR** |
| --- | --- | --- | --- | --- | --- | --- | --- |
| A | Time fight (%) P22-31 | t=0.7257, df=16 | 0.4785 | - | - | 100.0 | 148.7 |
| B | Time fight (%) P32-41 | t=2.856, df=18 | 0.0105 | - | - | 100.0 | 421.8 |
| C | Time fight (%) P42-51 | t=1.763, df=37 | 0.0909 | - | - | 100.0 | 49.60 |
| D | Time fight P22-31 | t=1.763, df=16 | 0.4785 | 9.464 | 14.07 | - | - |
| E | Time fight P32-41 | t=2.856, df=18 | 0.0105 | 19.31 | 81.46 | - | - |
| F | Time fight P42-51 | t=1.763, df=37 | 0.0909 | 59.11 | 29.32 | - | - |
| G | Biting P22-31 | t = 0.7731, df = 16 | 0.4507 | 43.51 | 66.4 | 4.118 | 6.284 |
| G | Rattling P22-31 | t = 0.7912, df = 16 | 0.4404 | 36.56 | 55.93 | 3.46 | 5.293 |
| G | Mounting P22-31 | t = 0.4843, df = 16 | 0.6347 | 19.93 | 26.93 | 1.887 | 2.493 |
| H | Biting P32-41 | t = 2.708, df = 18 | 0.0144 | 39.71 | 152.5 | 7.67 | 29.45 |
| H | Rattling P32-41 | t = 3.052, df = 18 | 0.0069 | 37.34 | 165.9 | 7.212 | 32.05 |
| G | Mounting P32-41 | t = 2.709, df = 18 | 0.0144 | 22.95 | 103.4 | 4.432 | 19.97 |
| I | Biting P42-51 | t = 1.665, df = 37 | 0.1043 | 39.42 | 20.08 | 23.3 | 11.87 |
| I | Rattling P42-51 | t = 1.674, df = 37 | 0.1026 | 36.81 | 19.06 | 21.76 | 11.27 |
| I | Mounting P42-51 | t = 1.887, df = 37 | 0.0670 | 23.76 | 10.45 | 14.05 | 6.18 |
| J | Latency P22-31 | Interaction F(2, 48)= 0.005262 | 0.9948 | Bite 834.4 | Bite 853.8 | - | - |
|  |  | Treatment F(1, 48)= 0.008338 | 0.9276 | Mount 863.3 | Mount 857.6 | - | - |
|  |  | Fight type F(2, 48)= 0.01602 | 0.9841 | Rattle 829.0 | Rattle 845.3 | - | - |
| K | Latency P32-41 | Interaction F(2, 54)= 0.02987 | 0.9706 | Bite 774.5 | Bite 603.1 | - | - |
|  |  | Treatment F(1, 54)= 4.134 | 0.0470 | Mount 785.9 | Mount 570.8 | - | - |
|  |  | Fight type F(2, 54)= 0.03804 | 0.9627 | Rattle 770.1 | Rattle 540.7 | - | - |
| L | Latency P42-51 | Interaction F(2, 111)= 0.002776 | 0.9972 | Bite 614.2 | Bite 778.2 | - | - |
|  |  | Treatment F(1, 111)= 3.454 | 0.0658 | Mount 624.1 | Mount 779.9 | - | - |
|  |  | Fight type F(2, 111)= 0.007083 | 0.9929 | Rattle 603.2 | Rattle 775.1 | - | - |
| M | Average bout P22-31 | Interaction F(2, 48)= 0.1328 | 0.8759 | Bite  6.711 | Bite  10.91 | - | - |
|  |  | Treatment F(1, 48)= 2.170 | 0.1473 | Mount  3.978 | Mount  5.778 | - | - |
|  |  | Fight type F(2, 48)= 1.414 | 0.2531 | Rattle  7.133 | Rattle  11.73 | - | - |
| N | Average bout P32-41 | Interaction F(2, 54)= 1.165 | 0.3197 | Bite  9.980 | Bite  24.18 | - | - |
|  |  | Treatment F(1, 54)= 15.55 | 0.0002 | Mount  6.520 | Mount  14.28 | - | - |
|  |  | Fight type F(2, 54)= 3.266 | 0.0458 | Rattle  11.10 | Rattle  32.58 | - | - |
| O | Average bout P42-51 | Interaction F(2, 111)= 0.06902 | 0.9334 | Bite  26.66 | Bite  15.47 | - | - |
|  |  | Treatment F(1, 111)= 5.188 | 0.0247 | Mount  15.57 | Mount  7.589 | - | - |
|  |  | Fight type F(2, 111)= 2.411 | 0.0944 | Rattle  28.89 | Rattle  17.05 | - | - |

**Table 2. Statistics for Supplementary Figure 2**

| **Panel** | **Groups comparison** | **Test result** | **P value** | **mean VEH VTA** | **mean GBR VTA** | **mean**  **VEH SNc** | **mean**  **GBR SNc** |
| --- | --- | --- | --- | --- | --- | --- | --- |
| A | Cells active per tract | Interaction F(1,33 )= 2.392 | 0.1315 |  |  |  |  |
|  |  | Treatment F(1, 33)= 6.753 | 0.0087 | 5.0 | 10.0 | 3.5 | 4.769 |
|  |  | Brain region F(1, 33)= 7.784 | 0.0139 |  |  |  |  |
| B | Firing rate | Interaction F(1, 203)= 0.06954 | 0.7923 |  |  |  |  |
|  |  | Treatment F(1, 203 )= 0.09833 | 0.7542 | 3.714 | 3.738 | 4.417 | 4.702 |
|  |  | Brain region F(1, 203 )= 2.856 | 0.0926 |  |  |  |  |
| C | Percent spike in burst | Interaction F(1, 171 )= 0.3133 | 0.5764 |  |  |  |  |
|  |  | Treatment F(1, 171)= 6.634 | 0.0109 | 17.03 | 35.14 | 21.91 | 33.55 |
|  |  | Brain region F(1, 171)= 0.0809 | 0.7764 |  |  |  |  |
| D | Burst firing rate | Interaction F(1, 174)= 0.6632 | 0.4165 |  |  |  |  |
|  |  | Treatment F(1, 174 )= 3.108 | 0.3678 | 19.57 | 22.58 | 14.11 | 22.30 |
|  |  | Brain region F(1, 174)= 0.8152 | 0.0796 |  |  |  |  |

**Table 3. Statistics for Supplementary Figure 3**

| **Panel** | **Groups comparison** | **Test result** | **P value** | **mean OFF** | **mean ON** | **mean OFF** | **mean ON** |
| --- | --- | --- | --- | --- | --- | --- | --- |
| A | Open field | Interaction F(3, 144)= 16.27 | <0.0001 |  |  |  |  |
|  | WT;Ai32 VS DatCre;Ai32 | Stimulation F(2.302, 110.5)= 18.88 | <0.0001 | WT 856.8 | WT 766.9 | WT 652.9 | WT 648.7 |
|  |  | Genotype F(1, 48)= 17.89 | 0.0001 | DatCre  1047 | DatCre  1403 | DatCre  876.1 | DatCre  1274 |

**Table 4. Statistics for Supplementary Figure 5**

**Table 5. Statistics for Supplementary Figure 6**

| **Panel** | **Groups comparison** | **Test result** | **P value** | **mean VEH** | **mean GBR** |
| --- | --- | --- | --- | --- | --- |
| A | Latency to lever press | t=0.6558, df=15 | 0.5219 | 1.365 | 1.213 |
| B | Latency to reward | t=1.939, df=18 | 0.0683 | 0.4420 | 0.3299 |

| **Panel** | **Groups comparison** | **Test result** | **P value** | **mean**  **No Stim WT** | **mean**  **Stim WT** | **mean**  **No Stim**  **DatCre** | **mean Stim DatCre** |
| --- | --- | --- | --- | --- | --- | --- | --- |
| A | Latency to lever press | Optogenetic effect F(3, 22)= 0.6365 | 0.5995 |  |  |  |  |
|  | WT;Ai32 VS DatCre;Ai32 |  |  | 0.9799 | 0.8831 | 0.9809 | 0.9836 |
| A | Latency to reward | Optogenetic effect F(3, 22)= 1.519 | 0.2375 |  |  |  |  |
|  | WT;Ai32 VS DatCre;Ai32 |  |  | 0.6537 | 0.6514 | 0.5101 | 0.5520 |
